# Health-related conditions among long-term cancer survivors diagnosed in adolescence and young adulthood (AYA): results of the SURVAYA study

**DOI:** 10.1007/s11764-024-01597-0

**Published:** 2024-05-13

**Authors:** Silvie H. M. Janssen, Carla Vlooswijk, Rhodé M. Bijlsma, Suzanne E. J. Kaal, Jan Martijn Kerst, Jacqueline M. Tromp, Monique E. M. M. Bos, Tom van der Hulle, Roy I. Lalisang, Janine Nuver, Mathilde C. M. Kouwenhoven, Winette T. A. van der Graaf, Olga Husson

**Affiliations:** 1 Department of Psychosocial Research and Epidemiology, Netherlands Cancer Institute, 1066 CX Amsterdam, the Netherlands; 2 Department of Medical Oncology, Netherlands Cancer Institute–Antoni van Leeuwenhoek, 1066 CX Amsterdam, the Netherlands; 3 Research and Development, Netherlands Comprehensive Cancer Organization, 3511 DT Utrecht, The Netherlands; 4 Department of Medical Oncology, University Medical Center Utrecht, 3584 CX Utrecht, The Netherlands; 5 Department of Medical Oncology, Radboud University Medical Center, 6525 GA Nijmegen, The Netherlands; 6 Department of Medical Oncology, Amsterdam University Medical Centers, 1105 AZ Amsterdam, The Netherlands; 7 Department of Medical Oncology, Erasmus MC Cancer Institute, Erasmus University Medical Center, 3015 GD Rotterdam, The Netherlands; 8 Department of Medical Oncology, Leiden University Medical Center, 2333 ZA Leiden, The Netherlands; 9 Division of Medical Oncology, Department of Internal Medicine, Maastricht UMC+ Comprehensive Cancer Center, GROW-School of Oncology and Reproduction, Maastricht University Medical Center+, 6229 HX Maastricht, The Netherlands; 10 Department of Medical Oncology, University Medical Center Groningen, 9713 GZ Groningen, The Netherlands; 11 Department of Neurology, Cancer Center Amsterdam, Amsterdam UMC, Amsterdam University Medical Centers, Location VUmc, 1081 HV Amsterdam, The Netherlands; 12 Department of Surgical Oncology, Erasmus MC Cancer Institute, Erasmus University Medical Center, 3015 GD Rotterdam, the Netherlands

**Keywords:** Adolescents and young adults, AYAs, Cancer, Survivorship, Health-related conditions, Population-based data

## Abstract

**Background:**

With 5-year survival rates > 85%, gaining insight into the long-term and late health-related conditions of cancer survivors diagnosed in adolescence and young adulthood is of utmost importance to improve their quantity and quality of survival. This study examined the prevalence of and factors associated with, patient-reported health-related conditions and their latency times among long-term adolescent and young adult (AYA) cancer survivors.

**Methods:**

AYA cancer survivors (5–20 years after diagnosis) were identified by the population-based Netherlands Cancer Registry (NCR), and invited to participate in the SURVAYA questionnaire study. Participants reported the prevalence and date of diagnosis of health-related conditions. Clinical data were retrieved from the NCR.

**Results:**

Three thousand seven hundred seventy-six AYA cancer survivors (response rate 33.4%) were included for analyses. More than half of the AYAs (58.5%) experienced health-related conditions after their cancer diagnosis, of whom 51.4% were diagnosed with two or more conditions. Participants reported conditions related to vision (15.0%), digestive system (15.0%), endocrine system (14.1%), cardiovascular system (11.7%), respiratory system (11.3%), urinary tract system (10.9%), depression (8.6%), hearing (7.4%), arthrosis (6.9%), secondary malignancy (6.4%), speech-, taste and smell (4.5%), and rheumatoid arthritis (2.1%). Time since diagnosis, tumor type, age at diagnosis, and educational level were most frequently associated with a health-related condition.

**Conclusions:**

A significant proportion of long-term AYA cancer survivors report having one or more health-related conditions.

**Implications for cancer survivors:**

Future research should focus on better understanding the underlying mechanisms of, and risk factors for, these health-related conditions to support the development and implementation of risk-stratified survivorship care for AYA cancer survivors to further improve their outcomes.

**Clinical trials registration:**

NCT05379387.

**Supplementary Information:**

The online version contains supplementary material available at 10.1007/s11764-024-01597-0.

## Introduction

Cancer survivors diagnosed in adolescence and young adulthood form a distinct group within the oncology population for whom (inter)national awareness has increased over time [1]. Adolescent and young adult (AYA) cancer patients are considered unique due to their spectrum of cancers, developmental life challenges, and supportive care needs that differ from cancer patients diagnosed in childhood or older adulthood [2]. Over the last decades, the overall cancer incidence has increased and the overall 5-year relative survival is currently exceeding 85%, leading to a rapidly growing group of AYA cancer survivors [3]. These survivors are at risk of long-term and late health-related conditions due to their cancer and cancer treatment for the remainder of their lives [4, 5].

Unfortunately, until now there has been a dearth of understanding of long-term and late health-related conditions and associated risk factors in cancer survivors diagnosed in adolescence and young adulthood, in contrast to the wealth of knowledge of these factors in survivors diagnosed in childhood [4, 5]. The number of pediatric oncology-oriented scientific publications, establishment of big lifetime cohorts (like the St. Jude LIFE’s Childhood cancer survivor cohort), and development of risk- and evidence-based survivorship guidelines represent the rich variety of (inter)national initiatives aiming to improve the outcomes of childhood cancer survivors [6]. In the Netherlands, this has led, among others, to evidence-based ‘LATER’-clinics, where cancer survivors diagnosed in childhood are followed up for years after their cancer diagnosis as they are at increased risk of a variety of health-related conditions compared to their peers [7].

For a long time, survivors of cancer diagnosed in adolescence and young adulthood were considered part of the pediatric or older adult cancer population and thus part of either pediatric or older adult-oriented research cohorts in which they were underrepresented. Due to the lack of AYA-specific research, AYAs formed an understudied cancer population. Furthermore, the results of pediatric or older adult survivorship-oriented research cannot be directly extrapolated to cancer survivors diagnosed in adolescence and young adulthood, as differences exist in tumor types and tumor biology, treatment exposure, age at exposure, and developmental life phase, impacting treatment adherence and clinical trial participation rates for example [2, 4, 5, 8–10]. Altogether, this has led to a lack of high-quality AYA-specific data and knowledge on long-term and late health-related conditions, impeding AYA-specific evidence- and risk-based survivorship guideline development [2, 11]. New insights can help to: better understand these conditions; support the development, implementation, and/or improvement of interventions; refer high-risk subgroups to the right interventions; and tailor survivorship care programs to screen for, prevent, and manage long-term and late health-related conditions*.* This knowledge and the use thereof may significantly impact the quantity and quality of AYAs’ survival*.*

The aims of this study were to examine (1) the prevalence of patient-reported health-related conditions among AYA cancer survivors, aged 18–39 years at primary cancer diagnosis according to the Dutch AYA age definition; (2) the latency time between these health-related conditions and cancer diagnosis; and (3) which AYA cancer survivor subgroups are at risk of developing health-related conditions based on sociodemographic and clinical characteristics.

## Methods

### Data collection

Data from the SURVAYA study, a retrospective, population-based, observational, cross-sectional cohort study, was used to analyze the health-related conditions of long-term cancer survivors diagnosed in adolescence and young adulthood (Clinical Trials Registration: NCT05379387) [12]. All AYA cancer survivors diagnosed with their first malignancy at the age of 18–39 years at one of the nine Dutch cancer centers and who have survived 5–20 years (age range at study participation: 23–60 years), were selected from the Netherlands Cancer Registry (NCR). Then, all records were linked to and checked against the Dutch municipal records database to verify whether AYAs were still alive at the moment of invitation and to obtain up-to-date addresses. As cancer care in the Netherlands is divided into pediatric oncology (< 18 years at cancer diagnosis) and medical oncology (≥ 18 years at cancer diagnosis), the lower age limit of the AYA definition applied in the SURVAYA study was set to 18 years.

Participants could complete the questionnaire either online or on paper. After completion, the questionnaire data were linked to the clinical data of the NCR to finalize the dataset. Approval of the linkage, access, and utilization of the clinical data was provided by the NCR, and the Institutional Review Board of the Antoni van Leeuwenhoek–Netherlands Cancer Institute reviewed and approved this study (IRBd18122).

### Measures

#### Sociodemographic characteristics

Sex (sex assigned at birth) was available from the NCR. Marital status, highest educational level achieved, and living status were self-reported by the participants.

#### Clinical characteristics

Type of cancer (classified according to the Third International Classification of Diseases for Oncology (ICDO-3)), age at and time since cancer diagnosis, type of primary treatment, and tumor stage (classified according to TNM or Ann Arbor Code) were available from the NCR.

#### Health-related conditions

Health-related conditions were assessed using a short, adapted version of the St. Jude Childhood Cancer Survivorship study questionnaire and self-reported by the participants [13]. In total, 12 health-related conditions (and their subsequent subconditions) were included in the questionnaire, which relates to (1) hearing, (2) vision, (3) speech, taste, and smell, (4) urinary tract system, (5) endocrine system, (6) cardiovascular system, (7) respiratory system, (8) digestive system, (9) rheumatoid arthritis (joint inflammation), (10) arthrosis (joint wear), (11) depression, and (12) secondary malignancy, of which the first eight consist of subconditions (Appendix Table 1). For each health-related condition (domain), participants could indicate whether they were diagnosed with it and, if so, with which subcondition(s) and when the subcondition was diagnosed (month and year). All health-related subconditions were categorized based on their date of diagnosis relative to the cancer diagnosis (patient level): health-related subconditions diagnosed more than 6 months after the cancer diagnosis were considered post-cancer diagnosis. Note that no direct causality between the diagnosis of the cancer and health-related condition can be proven. If any of the subconditions within a condition was diagnosed after cancer, the health-related condition would be categorized as post-diagnosis (yes vs no): an AYA could have multiple post-cancer subconditions (within one domain) which together counted as one condition for final analysis. The latency time was defined as the time in months between the date of the cancer diagnosis and the diagnosis of the health-related subcondition.

### Data analysis

Descriptive statistics were used to characterize the AYA cancer survivor population (frequencies and percentages for categorical data; means and standard deviations for continuous data). Chi-square tests (categorical data) and independent samples *t* tests (continuous data) were performed to compare included and excluded AYA cancer survivors (based on their (non-)response regarding the health-related conditions), and those with and without health-related conditions based on sociodemographic and clinical characteristics. To examine the association between sociodemographic and clinical characteristics and health-related conditions, 13 univariable and multivariable logistic regression analyses were performed: the first one in regards to having any health-related condition and the following 12 corresponding with the 12 health-related conditions (see 2.2.3). In case of no multicollinearity, independent variables with *p* < 0.1 in univariable logistic regression analyses were included in the multivariable logistic regression analyses. Multicollinearity was tested with tolerance, variance inflation factor, and variance proportion values. Missing data was not imputed and assumed missing at random. Two-sided *p* values of < 0.05 were considered statistically significant. IBM SPSS Statistics version 25 (SPSS Inc. Chicago, IL, USA) was used for all statistical analyses.

## Results

In total, 4010 AYA cancer survivors participated in the SURVAYA study [12], of which 234 did not complete any question regarding their health-related conditions and were therefore considered missing and excluded from the main analyses. The final cohort consisted of 3776 AYA cancer survivors (of 11,296 AYAs invited for study participation; a response rate of 33.4%). The complete recruitment procedure of the SURVAYA study has been described by Vlooswijk et al. [12]. AYAs who are female, treated with chemotherapy, diagnosed with a sarcoma, central nervous system tumor, or lymphoid malignancy, stage III or more than 10 years ago, were more likely to participate compared to their reference groups [12].

### Sociodemographic and clinical characteristics of the AYA cancer survivor population

Table 1 provides an overview of the sociodemographic and clinical characteristics of the included and excluded AYA cancer survivors. No significant differences were found between the included and excluded AYAs, except for age at diagnosis. Participating AYAs were on average 45 years old (standard deviation: 8) at questionnaire completion. The average time since cancer diagnosis was 12 years (standard deviation: 5).

**Table 1:** Sociodemographic and clinical characteristics of included vs excluded AYA cancer survivors

			AYA cancer survivors (*N* = 4010)				*p* value
			Included for further analysis		Excluded from further analysis		
			*N* = 3776 (94,2)		*N* = 234 (5,8)		
			*N*	%	*N*	%	
Age at diagnosis		18–24 years	586	15.5	27	11.5	0.02*
		25–34 years	1661	44.0	125	53.4	
		35–39 years	1529	40.5	82	35.0	
		Mean (SD) in years	31.6	5.9	31.5	5.4	
Time since diagnosis		< 11 years	1522	40.3	105	44.9	0.33
		11–15 years	1307	34.6	78	33.3	
		> 15 years	947	25.1	51	21.8	
		Mean (SD) in years	12.4	4.5	11.8	4.5	
Age at questionnaire completion		23–39	1018	27.0	71	30.3	0.42
		40–49	1805	47.8	111	47.4	
		50–60	953	25.2	52	22.2	
		Mean (SD) in years	44.5	7.5	43.8	7.2	
Sex		Male	1470	38.9	79	33.8	0.12
		Female	2306	61.1	155	66.2	
Partner status at the time of the questionnaire		Partner	3143	83.2	190	81.2	0.61
		No partner	620	16.4	41	17.5	
		Missing	13	0.3	3	1.3	
Education (achieved)		No education or primary education	24	0.6	4	1.7	0.20
		Secondary education	253	6.7	13	5.6	
		Secondary vocational education	1371	36.3	85	36.3	
		Higher (vocational) education	1286	34.1	88	37.6	
		University education	834	22.1	44	18.8	
		Missing	8	0.2	–	–	
Living status		Living alone	470	12.4	32	13.7	0.57
		Not living alone#	3298	87.3	201	85.9	
		Missing	8	0.2	1	0.4	
Tumor type		Head and neck	117	3.1	7	3.0	na
		Digestive track, other	30	0.8	1	0.4	
		Colon and rectal	76	2.0	6	2.6	
		Bone and soft tissue	166	4.4	6	2.6	
		Respiratory tract	27	0.7	3	1.3	
		Melanoma	265	7.0	25	10.7	
		Other	10	0.3	1	0.4	
		Germ cell	660	17.5	32	13.7	
		Breast	892	23.6	52	22.2	
		Female genitalia	409	10.8	36	15.4	
		Male genitalia	5	0.1	1	0.4	
		Urinary tract	41	1.1	5	2.1	
		Lymphoid hematological	561	14.9	30	12.8	
		Myeloid hematological	140	3.7	8	3.4	
		Thyroid gland	233	6.2	15	6.4	
		Central nervous system	144	3.8	6	2.6	
Primary treatment^	Chemotherapy	No	1650	43.7	117	50.0	0.06
		Yes	2122	56.2	117	50.0	
		Missing	4	0.1	–	–	
	Radiotherapy	No	1975	52.3	129	55.1	0.41
		Yes	1797	47.6	105	44.9	
		Missing	4	0.1	–	–	
	Endocrine therapy	No	3312	87.7	210	89.7	0.38
		Yes	460	12.2	24	10.3	
		Missing	4	0.1	–	–	
	Targeted therapy	No	3481	92.2	217	92.7	0.80
		Yes	291	7.7	17	7.3	
		Missing	4	0.1	–	–	
	Surgery	No	827	21.9	53	22.6	0.80
		Yes	2945	78.0	181	77.4	
		Missing	4	0.1	–	–	
	Stem cell therapy	No	3636	96.3	228	97.4	0.40
		Yes	136	3.6	6	2.6	
		Missing	4	0.1	–	–	
Tumor stage		I	1615	42.8	111	47.4	0.40
		II	1002	26.5	61	26.1	
		III	539	14.3	34	14.5	
		IV	173	4.6	6	2.6	
		Missing	447	11.8	22	9.4	
Health-related condition (any)		Yes	2208	58.5			
		No	1568	41.5			

*Bold*: statistically significant; *p < 0.05; **p < 0.01; *na* = not applicable

^#^Includes living with partner, parents, children, roommates or others

^ > 100% as participants may have received multiple treatments

### Prevalence of health-related conditions among AYA cancer survivors

**Fig. 1:**
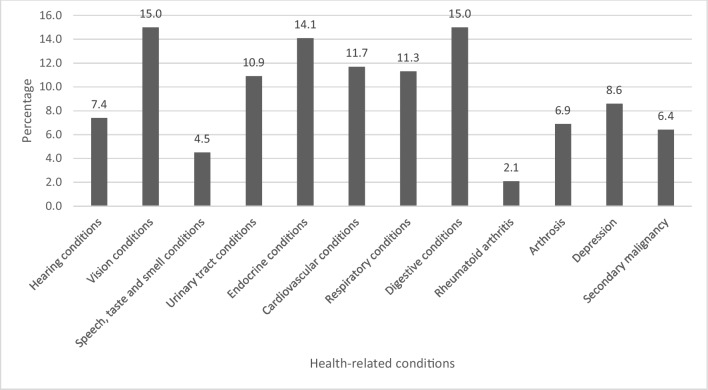
Prevalence of health-related conditions among AYA cancer survivors per condition

More than half of the participants (58.5%) experienced at least one health-related condition (any) (Appendix Table 2) post-cancer diagnosis, which could range from one to nine conditions. Of those with conditions, more than half (51.4%) had two or more conditions. Most often, AYAs had at least one health-related condition related to their vision (15.0%), digestive system (15.0%), endocrine system (14.1%), cardiovascular system (11.7%), respiratory system (11.3%) and urinary tract system (10.9%) (Fig. 1). Less common health-related conditions included depression (8.6%), hearing conditions (7.4%), arthrosis (6.9%), secondary malignancy (6.4%), speech, taste and smell conditions (4.5%), and rheumatoid arthritis (2.1%).

### Prevalence and latency time of health-related subconditions among AYA cancer survivors

**Fig. 2:**
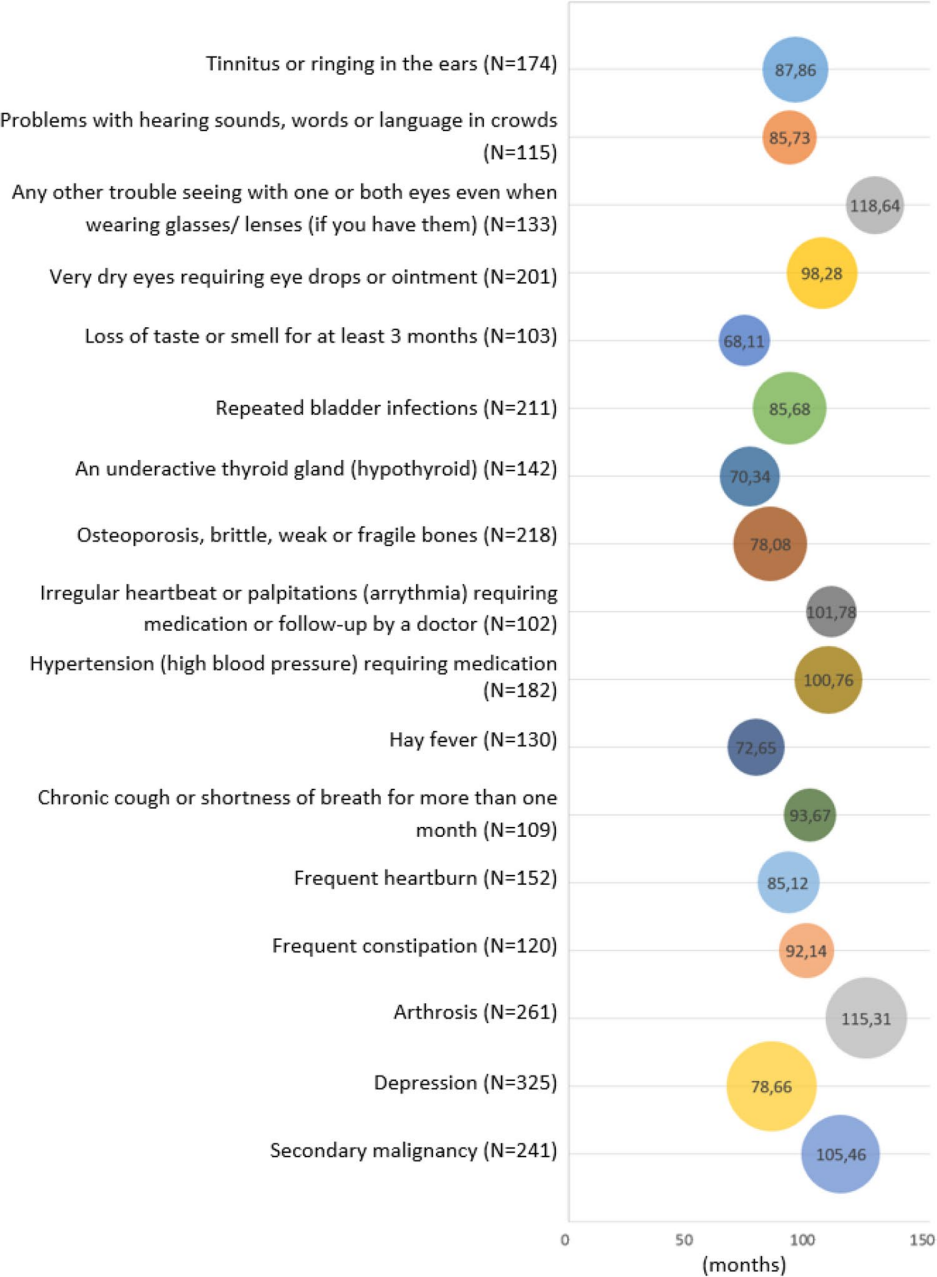
Prevalence (*N*) and average latency time (in months) per subcondition (*N* > 100) among long-term AYA cancer survivors. Note: the size of the circle represents the number of AYAs with that subcondition (a higher prevalence leads to a bigger circle)

Appendix Table 3 shows the number of AYAs and latency time per health-related subcondition. The most common subconditions included tinnitus and problems with hearing sounds, words, or language in crowds (*hearing*); trouble seeing with one or both eyes even when wearing glasses/lenses and very dry eyes requiring eye drops or ointment (*vision*); loss of taste or smell for at least 3 months (*speech, taste, and smell*); repeated bladder infections (*urinary tract system*); hypothyroidism and osteoporosis, brittle, weak or fragile bones (*endocrine system*); irregular heartbeat or palpitations (arrhythmia) requiring medication or follow-up by a doctor and hypertension requiring medication (*cardiovascular system*); hay fever and chronic cough or shortness of breath for more than 1 month (*respiratory system*); frequent heartburn and frequent constipation (*digestive system*); arthrosis; depression and a secondary malignancy diagnosis. The latency time of the subconditions ranged from 18.0 (complete deafness in both ears) to 155.2 months (narrowing or disease of the coronary artery). Figure 2 represents the prevalence and average latency time of the most common subconditions (*N* > 100).

### Associations between sociodemographic and clinical characteristics and health-related conditions among AYA cancer survivors

Due to signs of multicollinearity between age at questionnaire completion, age at diagnosis, and time since diagnosis, it prevented all three variables from being included. Therefore, age at questionnaire completion was excluded from all regression analyses. Factors significantly associated with a health-related condition included being diagnosed at the age of 35–39, female, 11–15 and > 15 years past one’s cancer diagnosis, diagnosed with ‘other’ tumor types, having completed secondary vocational education and higher (vocational) education, and having received stem cell therapy (Table 2). Survivors who underwent surgery were less likely to have health-related conditions, compared to those who did not undergo surgery.

**Table 2:** Associations between sociodemographic and clinical variables and health-related conditions among AYA cancer survivors (any condition)

			Total AYA population—health-related condition^$^ (*N* = 3776)					
			Univariable logistic regression			Multivariable logistic regression		
						Nagelkerkes *R*^2^ = 0.102		
			OR	95% CI	*p* value	OR	95% CI	*p *value
Age at diagnosis in years		18–24 years	REF			REF		
		25–34 years	0.969	0.802–1.172	0.749	0.992	0.808–1.218	0.939
		35–39 years	1.338	1.103–1.624	0.003	1.335	1.072–1.662	0.010
Time since diagnosis in years		< 11 years	REF			REF		
		11–15 years	1.632	1.405–1.896	< 0.001	1.747	1.494–2.042	< 0.001
		> 15 years	2.410	2.030–2.860	< 0.001	2.566	2.144–3.070	< 0.001
Sex		Male	REF			REF		
		Female	1.534	1.343–1.751	< 0.001	1.618	1.320–1.984	< 0.001
Partner status at the time of the questionnaire		Partner	0.823	0.689–0.982	0.031	0.921	0.726–1.169	0.501
		No partner	REF			REF		
Education (achieved)		No education or primary education	1.290	0.567–2.938	0.544	1.316	0.538–3.218	0.547
		Secondary education	1.342	1.009–1.785	0.043	1.121	0.831–1.513	0.455
		Secondary vocational education	1.556	1.307–1.853	< 0.001	1.445	1.203–1.735	< 0.001
		Higher (vocational) education	1.273	1.069–1.517	0.007	1.244	1.036–1.494	0.019
		University education	REF			REF		
Living status^#^		Living alone	1.280	1.048–1.564	0.016	1.209	0.922–1.586	0.169
		Not living alone#	REF					
Tumor type		Breast	REF			REF		
		Digestive track, other	1.285	0.595–2.779	0.523	2.144	0.928–4.951	0.074
		Colon and rectal	1.102	0.678–1.790	0.695	1.641	0.962–2.799	0.069
		Bone and soft tissue	0.761	0.545–1.063	0.109	1.099	0.734–1.645	0.647
		Respiratory tract	1.285	0.571–2.894	0.544	2.144	0.892–5.156	0.088
		Melanoma	0.583	0.442–0.768	< 0.001	0.903	0.619–1.318	0.597
		Other	2.571	0.543–12.177	0.234	4.998	1.017–24.574	0.048*
		Germ cell	0.590	0.482–0.724	< 0.001	1.119	0.796–1.573	0.517
		Head and neck	0.957	0.646–1.419	0.828	1.249	0.780–2.000	0.354
		Female genitalia	1.170	0.918–1.492	0.205	1.348	0.978–1.860	0.068
		Male genitalia	0.428	0.071–2.577	0.355	0.737	0.116–4.684	0.746
		Urinary tract	0.821	0.437–1.544	0.541	1.364	0.684–2.722	0.378
		Lymphoid hematological	1.255	1.007–1.565	0.044	1.022	0.621–1.682	0.930
		Myeloid hematological	1.357	0.929–1.983	0.115	0.776	0.414–1.455	0.429
		Thyroid gland	0.917	0.684–1.230	0.564	1.338	0.925–1.934	0.122
		Central nervous system	0.739	0.518–1.052	0.093	1.288	0.839–1.977	0.246
Primary treatment	Chemotherapy	No	REF			REF		
		Yes	1.276	1.120–1.454	< 0.001	1.130	0.934–1.366	0.208
	Radiotherapy	No	REF			REF		
		Yes	1.307	1.148–1.489	< 0.001	1.114	0.947–1.310	0.195
	Endocrine therapy	No	REF			REF		
		Yes	1.230	1.006–1.504	0.044	1.163	0.881–1.536	0.286
	Targeted therapy	No	REF					
		Yes	0.952	0.748–1.213	0.693			
	Surgery	No	REF			REF		
		Yes	0.608	0.517–0.716	< 0.001	0.555	0.369–0.836	0.005
	Stem cell therapy	No	REF			REF		
		Yes	2.963	1.934–4.538	< 0.001	2.571	1.597–4.138	< 0.001
Tumor stage		I	REF					
		II	1.104	0.941–1.295	0.227			
		III	1.007	0.827–1.226	0.943			
		IV	1.175	0.853–1.620	0.323			

No multicollinearity was found for any of the independent variables [multicollinearity is indicated by: tolerance value < 0.1, variance inflation factor value > 10 and variance proportions ≥ 0.7 on the same eigenvalue]

*Should be interpreted with caution due to wide 95% confidence intervals; *REF* reference group; *CI* confidence interval; *OR* Odds ratio; # Includes living with partner, parents, children, roommates, or others

^$^The number of AYAs who are diagnosed with a new primary might be underestimated as we have recategorized and added the answering option ‘I don’t know’ to ‘No’

Multivariable logistic regression per health-related condition showed that tumor type was significantly associated with speech, taste and smell, urinary tract, cardiovascular, respiratory, and digestive conditions, rheumatoid arthritis, depression, and secondary malignancies (Appendix Table 4a-l). Radiotherapy was significantly associated with cardiovascular, respiratory, and digestive conditions. Endocrine therapy and surgery were both significantly associated with endocrine conditions. In regard to sociodemographic factors, age at diagnosis was significantly associated with endocrine, cardiovascular, and respiratory conditions, arthrosis, depression, and secondary malignancies. Time since diagnosis was significantly associated with all conditions except for conditions related to speech, taste and smell, and depression. Living status was significantly associated with cardiovascular conditions and partner status was significantly associated with urinary tract conditions and depression. Sex was only significantly associated with hearing and endocrine conditions, while education was significantly associated with endocrine and cardiovascular conditions, rheumatoid arthritis, arthrosis, and depression.

## Discussion

We aimed to describe the prevalence, latency time, and associated factors of health-related (sub)conditions among a unique cohort of 3776 cancer survivors diagnosed in adolescence and young adulthood with different cancer diagnoses and a follow-up of 5–20 years since primary cancer diagnosis. Our results show that more than half of the participants have at least one health-related condition and that 51.4% of them report multiple conditions. The most common conditions relate to their vision or digestive, endocrine, cardiovascular, respiratory, and urinary tract systems. Large variances were found in the average latency times between subconditions, ranging from 18 (complete deafness in both ears) to 155 months (narrowing or disease of the coronary artery). Older age at cancer diagnosis, longer time since cancer diagnosis, secondary vocational and higher (vocational) education, and tumor types like female genitalia and thyroid malignancies were most often significantly associated with health-related conditions (compared to their reference categories). Our results also show that several health-related subconditions, such as osteoporosis, were more frequently reported which requires more in-depth analyses.

Several results of our study are in line with the findings of previous studies. Almost two out of three survivors (more than 5 years after diagnosis, range of cancer types) reported at least one health-related condition, similar to studies previously performed by Mellblom et al., Abdelhadi et al., and Oeffinger et al. [14–16]. The variety in latency times and conditions among organs and systems that develop alongside each other (having multiple health-related conditions) is also well known [14, 16, 17]. More prevalent health-related conditions shown in our study, including conditions related to the digestive [18], endocrine [5, 15, 16, 18], cardiovascular [5, 14, 16, 18], respiratory [16], and urinary tract system [14], were also commonly reported in other studies. The study of Tai et al. demonstrated that 14% of their AYA cancer survivors were diagnosed with cardiovascular diseases, which is comparable to our results (11.7%) [19]. In addition, the likelihood of developing health-related conditions differs based on sociodemographic and clinical characteristics. For example, overall it was shown that survivors who underwent surgery were less likely to have health-related conditions compared to those who did not undergo surgery, as opposed to those who underwent radiotherapy (compared to no radiotherapy) [5, 18]. Surgeries are overall much more local and the tumor is often resected, while therapies like radiotherapy can affect a wider physical area, which might explain the difference in reported health-related conditions.

However, study results are not always in accordance. The review of Ryder-Burbidge [4] describes inconsistent results regarding the associations between the risk of subsequent malignant neoplasms with sex and age at diagnosis: our results showed no significant association with sex. However, older age at primary cancer diagnosis was significantly associated with an increased likelihood of developing secondary malignancies. Similarly, in the study of Abdelhadi et al., 6.4% of the AYAs were diagnosed with bronchitis compared to 1.2% in our study [16]. Differences between study results might be explained by the diversity in study characteristics [17]. Oeffinger et al. included younger cancer patients (< 21 years at cancer diagnosis), more time has passed since their cancer diagnosis (6–31 years since cancer diagnosis), and the range of included tumor types was more restricted (leukemia, central nervous system tumors, Hodgkin’s disease, non-Hodgkin’s lymphoma, Wilm’s tumor, neuroblastoma, sarcoma, and bone tumors) compared to our study [14]. Chao et al. included survivors who were also more recently diagnosed (2–15 years since cancer diagnosis) and data was collected using electronic medical records [5]. The range of health-related conditions differed compared to ours, as outcomes such as anxiety and ovarian failure were also covered. Tai et al. included younger cancer patients (15–29 years at cancer diagnosis), and data was collected through telephone interviews [19]. Although the range of time since diagnosis was broader (5–30 years since cancer diagnosis), the range of tumor types covered in the study of Mellblom et al. was limited to breast cancer, colorectal cancer, non-Hodgkin lymphoma, leukemia, and melanoma [15]. The range of health-related conditions covered outcomes such as fatigue, concentration/memory problems, and psychological reactions, which were not fully covered in our study. Making comparisons between (international) studies is limited by the large variation in methods, as also highlighted by Woodward et al. [20].

Several treatment and tumor-specific associations with health-related conditions were found, including (1) head and neck cancer survivors had a significantly increased likelihood of reporting conditions related to speech, taste and smell, (2) respiratory system conditions were significantly more likely to be reported by AYAs with respiratory tract malignancies, (3) survivors of female genitalia, urinary tract, and thyroid gland malignancies were significantly more likely to report urinary tract conditions, and (4) AYAs diagnosed with colorectal, other digestive tract and female genitalia malignancies were significantly more likely to have digestive system conditions. Although some associations are less self-evident, most of the abovementioned associations are as expected as the health-related condition and location of the tumor refer to the same system or are physically close to each other. The association found between stem cell therapy and a health-related condition [4] is well-known and related to the inherent toxicity of the treatment. As expected, AYAs who were treated with endocrine therapy were more likely to report conditions of the endocrine system. Although the abovementioned health-related conditions and associations are not age-specific per se, the impact, on the other hand, can be. As AYAs are in the prime of their life and have a long life ahead of them, experiencing conditions can have a significant impact on various aspects of their daily life (family, social, education, employment, psychological, independence), and the quality thereof [2, 21]. Current cancer survivorship care is not yet always aligned with the age-specific needs of AYA cancer survivors, especially in the long term [2, 22]. Evidence-based, risk-stratified, AYA-focused survivorship guidelines are needed, which delineate care pathways, responsible stakeholders, and reimbursement options among others. It should be noted, however, that treatments have changed over time, potentially leading to changes in the prevalence and types of health-related conditions. With the advent of new therapies such as immunotherapy, which were not represented in our study among long-term survivors, future research should inform us about health-related conditions caused by these new therapies.

Our results show that several health-related conditions were associated with a survivor’s partner status, educational level, and living situation. Having a partner was associated with a reduced likelihood of having depression for example, and those living alone were more likely to have cardiovascular conditions. It could be that AYAs with a close support system live healthier as they have someone by their side to support them or they themselves are responsible for the care of others, like their children. Previous research has highlighted the positive association between social support and health behaviors, such as smoking, physical activity, and eating behavior, and health-related outcomes, such as quality of life [23–27]. In line with this, recent studies have also identified so-called social determinants of health (SDOH), defined as non-medical factors that can influence health outcomes, such as access to housing, an adequate income, and health insurance [28–31]. SDOH might be of utmost relevance for AYAs, as they are well-known to be a vulnerable population due to their developmental life phase [32]. In order to adequately address health inequalities in this survivor population, future research should take into account SDOH, including lifestyle factors for example. In addition, as our study included mostly Caucasian and higher-educated AYAs, a better representation of subgroups (based on SDOH) should be an aim. This can potentially be realized by the use of objective/real-world data, defined as routinely generated data (excluding experimentally generated data), such as data from electronic health records and disease registries [33, 34]. Studies based on real-world data may avoid recall bias and participation burden, and improve generalization of study results [33, 34]. Finally, as this study represents a cross-sectional study design, no causal relationships can be established: it is recommended that future studies apply different methodological designs, preferably in a prospective setting.

Although most health-related conditions focused on physical outcomes, depression was the only condition representing the psychological burden among long-term AYA cancer survivors. AYAs who were older at their cancer diagnosis were significantly less likely to report depression than younger AYAs: this indicates that the timing of the cancer diagnosis can lead to a different psychological burden. Older AYAs might be in a more stable phase in their life when diagnosed and have more life experience, which may allow them to better cope with their new situation. It also emphasizes the importance of age-specific care in general, including psychological support. Previous research has also shown depression to be a common health-related condition among AYAs [35–37]: the added value of our study is reflected in the long-term follow-up of many survivors, showing that a psychological condition can still arise years after diagnosis (on average more than 6 years post-cancer diagnosis in our study). Both the prevalence and latency time underline the need for age-specific, psychosocial survivorship care, which is also expressed by AYA cancer survivors in previous research [1].

The highly frequent association between the variable time since diagnosis and the presence of health-related conditions is in line with our expectations: simply more time has passed in order for conditions to develop and there has been more exposure time to additional carcinogenic factors in life as AYAs age. It is, however, unclear whether these health-related conditions are due to regular biological aging or potentially caused by the mechanism of accelerated aging. The latter would suggest that the cancer and its treatment have accelerated the *normal* aging process in cancer survivors by speeding up the accumulation of cellular damage that leads to the premature development of certain conditions, leading to a higher biological than chronological age [38–40]. Although our results provide insight into the burden of health-related conditions, the design of our study, unfortunately, prevents us from making any conclusions regarding accelerated aging: data regarding sociodemographics and health-related conditions in a matched control population would be needed to analyze any significant effect of the cancer (treatment) specifically. In addition, validated aging biomarker data, for example from blood, are needed to track changes over time or between study populations. The COMPRAYA study, a large prospective observational cohort study, is a research initiative that is currently collecting this type of data [41].

### Clinical implications

The prevalence of and factors associated with health-related (sub)conditions underscore the importance of timely, tailored, age-specific survivorship care for a growing survivorship population who have a long life ahead of them. Different stakeholders, including AYAs and healthcare providers, should be made aware of and educated about health-related conditions that can develop during survivorship. This will hopefully (a) improve access to, provision of, and use of tailored care, (b) lower the burden of health-related conditions and (c) even possibly prevent some health-related conditions from developing at all. Besides creating awareness, it is important that age-specific survivorship guidelines are developed, clearly describing what type of healthcare should be available and provided to this unique population. Tailored interventions that take into account the timing, subgroups at risk, mode of delivery, costs, and other aspects that might impact their effectiveness, should be developed to put guidelines into practice in order to prevent and manage health-related conditions. This should ensure the quantity and quality of AYAs’ survival. In order to be able to provide the best age-specific care and support AYAs in the best possible way, it is imperative to educate healthcare providers about the characteristics of and health-related conditions that might develop among this population, and the impact thereof. Several studies have shown a substantial increase in healthcare use and medical expenses among AYA cancer survivors with (multiple) chronic conditions compared to control populations [4, 16, 31], stressing the importance of getting appropriate care at the right time, as is in line with our recommendations. Current pediatric cancer survivorship care may act as an example of how to develop and implement AYA cancer survivorship care, as it is overall well embedded into healthcare systems. It provides a great opportunity to learn from each other, without needing to reinvent the wheel. For this, close collaboration between pediatric and adult oncology is of utmost importance, as well as on an international scale [42]. Once survivorship guidelines are established and put into practice, quality indicators are needed to measure the provided survivorship care and indicate areas of improvement. Although our study provides more insight into which health-related (sub)conditions can arise as well as into the latency time and associated factors, more research is needed (including real-world data) in order to properly organize survivorship care. This should focus on which conditions arise and when, and through which mechanisms, as well as consider the presence of associated risk factors. Preferably this should be done by performing large-scale, AYA-specific studies that include the full AYA age range, have long-term follow-up (more than 5 years), and lead to risk-stratified results [4, 5].

### Strengths and limitations

The strengths and uniqueness of this cohort study include the very large sample size, long-term (more than 5 years) follow-up, population-based data, a broad range of cancer types, the rich dataset of many health-related (sub)conditions (prevalence and latency time) covering major organ systems, and use of an existing questionnaire that is also used in other study populations.

However, there are also some limitations. Firstly, no matched control population could be defined for which similar data regarding their health-related conditions was available, in order to define the causal effect of a cancer diagnosis and subsequent treatment. Secondly, no data is available regarding the severity and impact of any health-related condition. Therefore, no conclusions can be drawn regarding differences in impact between the health-related conditions, and how these may affect and challenge daily life. Thirdly, the health-related subconditions are self-reported and therefore they were not clinically verified: we rely on the participant’s memory and correct interpretation of all diagnoses’ names and dates, potentially introducing recall bias. In addition, if someone experiences hardly any/no symptoms and/or does not visit a healthcare provider, and is therefore possibly not aware of any conditions, then he/she could have a health-related condition that is not officially diagnosed and thus not recorded in this dataset. It is therefore assumed that the burden of health-related conditions is actually larger than currently validated and we possibly underestimate the impact. This might also partly be caused by the choice of categorization: if any (regardless how many) of the subconditions among a condition were categorized as post-cancer, then this was indicated in the dataset as having a health-related condition (either yes or no). Having a post-cancer diagnosis condition could thus represent one subcondition, but also multiple subconditions (even up to 18 subconditions in the digestive system). In addition, we might have underestimated the burden of health-related conditions as prognosis between cancer types differs and our results are possibly biased by the survivorship of AYAs who were relatively healthy or possibly received less intense treatment for example (survivorship bias). In line with this, the generalization of this study might be affected due to the lack of outcomes of non-participating AYAs [12]. Some subgroups were very small in numbers, which impacted the 95% confidence intervals. Also, the applied statistical methods may have impacted the interpretation of our study data [43]. The inclusion of the variables age at diagnosis, time since diagnosis, and age at questionnaire completion in the regression model was limited due to signs of multicollinearity. Due to the exclusion of age at questionnaire completion, the reported effects of age at diagnosis and time since diagnosis cannot be clearly distinguished from effects of age at questionnaire completion, and they do not provide information about the role of age at questionnaire completion in the observed relationships. Lastly, the current analysis is limited by covering only one psychological health-related condition (depression), and therefor lacks input regarding other psychological conditions that might result from cancer and cancer treatment.

## Conclusion

A significant proportion of long-term cancer survivors diagnosed in adolescence and young adulthood report the occurrence of one or more health-related condition(s), which cover a range of health systems, such as the digestive, cardiovascular, and endocrine systems. Associations with health-related conditions differed based on sociodemographic and clinical data: time since diagnosis, tumor type, age at diagnosis, and educational level were most frequently associated with a health-related condition in our study. Although our study provides an important insight into the prevalence of and factors associated with health-related conditions, future research should focus on better understanding the underlying mechanisms of these health-related conditions, and take into account a broader range of potentially associated factors, such as lifestyle factors and social determinants of health. These new insights should support the development and implementation of risk-stratified survivorship care for AYA cancer survivors in order to ensure the quantity and quality of their survival.

## Supplementary Information

Below is the link to the electronic supplementary material.

Supplementary file1 (DOCX 18 KB)

Supplementary file2 (DOCX 19 KB)

Supplementary file3 (DOCX 33 KB)

Supplementary file4 (DOCX 87 KB)

## Data Availability

The dataset generated and analyzed in this study is available from the corresponding author on reasonable request. The data are not publicly available due to privacy issues.
