# Supplementary material for: Health-related conditions among long-term cancer survivors diagnosed in adolescence and young adulthood (AYA): results of the SURVAYA study
Source: J Cancer Surviv. 2024 May 13;19(6):1821–34. doi: 10.1007/s11764-024-01597-0 (PMC12546281; doi:10.1007/s11764-024-01597-0)
Supplement: Supplementary file 1 — Supplementary file1 (DOCX 18 KB) [file 11764_2024_1597_MOESM1_ESM.docx]

**Appendix Table 1. Overview of the health-related (sub)conditions**

| **Condition** | **Subcondition** |
| --- | --- |
| **Hearing conditions** | Hearing loss (hearing impairment requiring a hearing aid) |
|  | Deafness in one or both ears (not completely corrected by hearing aid) |
|  | Complete deafness in both ears |
|  | Tinnitus or ringing in the ears |
|  | Persistent dizziness or vertigo |
|  | Problems with hearing sounds, words or language in crowds |
|  | Any other hearing condition |
| **Vision conditions** | Blind (in one or both eyes) |
|  | Cataract |
|  | Glaucoma (excess pressure in the eyeball) |
|  | Double vision |
|  | A detached retina or any other condition of the retina |
|  | Any other trouble seeing with one or both eyes even when wearing glasses/ lenses (if you have them) |
|  | Very dry eyes requiring eye drops or ointment |
|  | Any other eye condition |
| **Speech, taste and smell conditions** | Stammering or stuttering |
|  | Any other speech condition |
|  | Abnormal sense of taste |
|  | Loss of taste or smell for at least 3 months |
| **Urinary tract conditions (bladder, kidneys)** | Kidney stones |
|  | Repeated kidney infections (so-called pyelonephritis) |
|  | Repeated bladder infections |
|  | *Kidney dialysis* |
|  | Any other kind of kidney or urinary tract condition |
|  | *Kidney transplant* |
| **Endocrine conditions** | An overactive thyroid gland (hyperthyroid) |
|  | An underactive thyroid gland (hypothyroid) |
|  | Thyroid nodule |
|  | Thyroid enlargement |
|  | Diabetes - controlled with diet |
|  | Diabetes - controlled with pills or tablets, but not with insulin shots |
|  | Diabetes - controlled with (amongst others) insulin shots |
|  | Deficiency of growth hormone |
|  | *Have you ever received injections of growth hormone (such as somatropine)* |
|  | Osteoporosis, brittle, weak or fragile bones |
|  | Any other endocrine condition |
|  | *Removal of the thyroid gland* |
| **Cardiovascular conditions** | Rheumatic heart disease |
|  | Hardening of the arteries or arteriosclerosis |
|  | Irregular heartbeat or palpitations (arrythmia) requiring medication or follow-up by a doctor |
|  | Congestive heart failure or cardiomyopathy (weak heart muscle) |
|  | Myocardial infarction (heart attack) |
|  | Narrowing or disease of the coronary artery |
|  | Hypertension (high blood pressure) **not** requiring medication |
|  | Hypertension (high blood pressure) requiring medication |
|  | A stroke such as a TIA (Transient Ischemic Attack), cerebrovascular accident (CVA), or hemorrhagic stroke |
|  | Angina pectoris (chest pains due to lack of oxygen to heart) requiring medication such as nitroglycerine |
|  | Pericarditis or fluid around the heart |
|  | Pericardial constriction (scarring or constricting of the sac around the heart) |
|  | Stiff or leaking heart valves |
|  | Blood clot in head, lung, arm, leg or pelvis (thrombosis or embolism) |
|  | *Have you seen a cardiologist (heart specialist)? (fill in date of the first time)* |
|  | Any other heart or circulatory problem |
|  | *Have you ever had a coronary artery bypass surgery?* |
|  | *Have you ever had a pericardiectomy (stripping of the sac around the heart)?* |
|  | *Have you ever had an angioplasty (enlarging a heart vessel using a balloon)?* |
|  | *Have you ever had any other heart surgery?* |
|  | *Have you ever had a surgery to remove a blood clot in an artery or vein?* |
|  | *Has anyone in your immediate family (mother, father, brothers, sisters) had a heart attack before the age of 55?* |
| **Respiratory conditions** | Bronchitis |
|  | Hay fever |
|  | Recurrent sinus infection |
|  | Tonsillitis or enlargement of the tonsils or adenoids |
|  | Pleurisy (inflammation of the lining of the lungs) |
|  | Asthma |
|  | Abnormal chest wall |
|  | Chronic cough or shortness of breath for more than one month |
|  | *Have you ever had a need for extra oxygen?* |
|  | Pneuomania, 3 or more times in the past 2 years |
|  | Emphysema (COPD) |
|  | Lung fibrosis or "scarring" of the lung |
|  | Any other breathing or lung problem |
|  | *Have you ever had a bronchoscopy?* |
|  | *Have you ever had a lung transplant?* |
|  | *Have you ever had another lung surgery?* |
| **Digestive conditions** | Gallstones |
|  | Any other gallbladder condition |
|  | Cirrhosis of the liver |
|  | Hepatitis (inflammation of the liver, such as Hepatitis A, B, C, D, E) |
|  | Jaundice |
|  | Any other liver condition |
|  | A stomach ulcer |
|  | A condition of the esophagus |
|  | Frequent stomach pain (dyspepsia) |
|  | Frequent heartburn |
|  | Any other stomach condition |
|  | Intestinal polyps |
|  | Diverticular disease |
|  | Colitis (such as Chrohn's disease or ulcerative colitis) |
|  | Frequent constipation |
|  | Chronic diarrhea (more than 3 weeks ánd more than 3 days per week ánd more than 3 times per day) |
|  | Rectal or anal fistula |
|  | Rectal or anal stricture (narrowing or scarring) |
|  | *Have you ever had a surgery for intestinal obstruction (blocked intestines)?* |
|  | *Have you ever had a colostomy or ileostomy (stool going into a bag)?* |
|  | *Have you ever had a takedown (reconnection) of the colostomy or ileostomy?* |
|  | *Have you ever had a removal of the spleen?* |
|  | *Have you ever had a liver biopsy since your cancer therapy stopped?* |
| ***Anemia*** |  |
| **Rheumatoid arthritis** |  |
| **Arthrosis** |  |
| **Depression** |  |
| ***Back problems*** |  |
| ***Any other medical/ mental problem (1)*** | |
| ***Any other medical/ mental problem (2)*** | |
| ***Any other medical/ mental problem (3)*** | |
| **At any time following your original diagnosis, were you diagnosed with another cancer?** | |
|  | No |
|  | *Yes, recurrence or metastasis* |
|  | Yes, secondary malignancy |
|  | Don't know |
| *Note: red/italic indicates the item was not included in the final analysis, as these items refer to for example treatments or visits to a healthcare provider* | |
