# Supplementary material for: Health-related conditions among long-term cancer survivors diagnosed in adolescence and young adulthood (AYA): results of the SURVAYA study
Source: J Cancer Surviv. 2024 May 13;19(6):1821–34. doi: 10.1007/s11764-024-01597-0 (PMC12546281; doi:10.1007/s11764-024-01597-0)
Supplement: Supplementary file 2 — Supplementary file2 (DOCX 19 KB) [file 11764_2024_1597_MOESM2_ESM.docx]

**Appendix Table 2. Sociodemographic and clinical characteristics of AYAs with vs without any health-related conditions**

|  |  |  | **Included AYA cancer survivors (N=3776)** | | | |  |
| --- | --- | --- | --- | --- | --- | --- | --- |
|  |  |  | **AYAs with at least 1 condition** | | **AYAs with no conditions** | |  |
|  |  |  | **N= 2208**  **(58,5%)** | | **N= 1568**  **(41,5%)** | |  |
|  |  |  | **N** | **%** | **N** | **%** | **p-value** |
| Age at diagnosis | | 18-24 years | 328 | 14,9 | 258 | 16,5 | **<0,001** |
|  |  | 25-34 years | 917 | 41,5 | 744 | 47,4 |  |
|  |  | 35-39 years | 963 | 43,6 | 566 | 36,1 |  |
|  |  | Mean(SD) in years | 31,9 | 5,9 | 31,1 | 5,9 |  |
| Time since diagnosis |  | <11 years | 747 | 33,8 | 775 | 49,4 | **<0,001** |
|  |  | 11-15 years | 799 | 36,2 | 508 | 32,4 |  |
|  |  | >15 years | 662 | 30,0 | 285 | 18,2 |  |
|  |  | Mean(SD) in years | 13,1 | 4,5 | 11,5 | 4,4 |  |
| Age at questionnaire completion | | 23-39 | 513 | 23,2 | 505 | 32,2 | **<0,001** |
|  |  | 40-49 | 1006 | 45,6 | 798 | 50,9 |  |
|  |  | 50-60 | 689 | 31,2 | 265 | 16,9 |  |
|  |  | Mean(SD) in years | 45,6 | 7,6 | 43,1 | 7,1 |  |
| Sex |  | Male | 766 | 34,7 | 704 | 44,9 | **<0,001** |
|  |  | Female | 1442 | 65,3 | 864 | 55,1 |  |
| Partner status at time of questionnaire | | Partner | 1815 | 82,2 | 1328 | 84,7 | **0,03** |
|  |  | No partner | 387 | 17,5 | 233 | 14,9 |  |
|  |  | Missing | 6 | 0,3 | 7 | 0,4 |  |
| Education (achieved) | | No education or primary education | 14 | 0,6 | 10 | 0,6 | **<0,001** |
|  |  | Secondary education | 150 | 6,8 | 103 | 6,6 |  |
|  |  | Secondary vocational education | 861 | 39,0 | 510 | 32,5 |  |
|  |  | Higher (vocational) education | 746 | 33,8 | 540 | 34,4 |  |
|  |  | University education | 434 | 19,7 | 400 | 25,5 |  |
|  |  | Missing | 3 | 0,1 | 5 | 0,3 |  |
| Living status | Living alone |  | 299 | 13,5 | 171 | 10,9 | **0,02** |
|  | Not living alone# |  | 1904 | 86,2 | 1394 | 88,9 |  |
|  | Missing |  | 5 | 0,2 | 3 | 0,2 |  |
| Tumortype | Head and neck |  | 70 | 3,2 | 47 | 3,0 | na |
|  | Digestive track, other | | 20 | 0,9 | 10 | 0,6 |  |
|  | Colon and rectal |  | 48 | 2,2 | 28 | 1,8 |  |
|  | Bone and soft tissue | | 90 | 4,1 | 76 | 4,8 |  |
|  | Respiratory tract |  | 18 | 0,8 | 9 | 0,6 |  |
|  | Melanoma |  | 126 | 5,7 | 139 | 8,9 |  |
|  | Other |  | 8 | 0,4 | 2 | 0,1 |  |
|  | Germ cell |  | 316 | 14,3 | 344 | 21,9 |  |
|  | Breast |  | 543 | 24,6 | 349 | 22,3 |  |
|  | Female genitalia |  | 264 | 12,0 | 145 | 9,2 |  |
|  | Male genitalia |  | 2 | 0,1 | 3 | 0,2 |  |
|  | Urinary tract |  | 23 | 1,0 | 18 | 1,1 |  |
|  | Lymphoid hematological | | 371 | 16,8 | 190 | 12,1 |  |
|  | Myeloid hematological | | 95 | 4,3 | 45 | 2,9 |  |
|  | Thyroid gland |  | 137 | 6,2 | 96 | 6,1 |  |
|  | Central nervous system | | 77 | 3,5 | 67 | 4,3 |  |
| Primary treatment^ | Chemotherapy | No | 910 | 41,2 | 740 | 47,2 | **<0,001** |
|  |  | Yes | 1296 | 58,7 | 826 | 52,7 |  |
|  |  | Missing | 2 | 0,1 | 2 | 0,1 |  |
|  | Radiotherapy | No | 1094 | 49,5 | 881 | 56,2 | **<0,001** |
|  |  | Yes | 1112 | 50,4 | 685 | 43,7 |  |
|  |  | Missing | 2 | 0,1 | 2 | 0,1 |  |
|  | Endocrine therapy | No | 1917 | 86,8 | 1395 | 89,0 | **0,05** |
|  |  | Yes | 289 | 13,1 | 171 | 10,9 |  |
|  |  | Missing | 2 | 0,1 | 2 | 0,1 |  |
|  | Targeted therapy | No | 2039 | 92,3 | 1442 | 92,0 | 0,71 |
|  |  | Yes | 167 | 7,6 | 124 | 7,9 |  |
|  |  | Missing | 2 | 0,1 | 2 | 0,1 |  |
|  | Surgery | No | 559 | 25,3 | 268 | 17,1 | **<0,001** |
|  |  | Yes | 1647 | 74,6 | 1298 | 82,8 |  |
|  |  | Missing | 2 | 0,1 | 2 | 0,1 |  |
|  | Stem cell therapy | No | 2097 | 95,0 | 1539 | 98,2 | **<0,001** |
|  |  | Yes | 109 | 4,9 | 27 | 1,7 |  |
|  |  | Missing | 2 | 0,1 | 2 | 0,1 |  |
| Tumor stage |  | I | 917 | 41,5 | 698 | 44,5 | 0,53 |
|  |  | II | 593 | 26,9 | 409 | 26,1 |  |
|  |  | III | 307 | 13,9 | 232 | 14,8 |  |
|  |  | IV | 105 | 4,8 | 68 | 4,3 |  |
|  |  | Missing | 286 | 13,0 | 161 | 10,3 |  |
| Number of health-related conditions | | 1 health-related condition | 1073 | 48,6 |  |  |  |
|  |  | 2 health-related conditions | 603 | 27,3 |  |  |  |
|  |  | 3 health-related conditions | 280 | 12,7 |  |  |  |
|  |  | 4 health-related conditions | 143 | 6,5 |  |  |  |
|  |  | 5 health-related conditions | 68 | 3,1 |  |  |  |
|  |  | 6 health-related conditions | 23 | 1,0 |  |  |  |
|  |  | 7 health-related conditions | 11 | 0,5 |  |  |  |
|  |  | 8 health-related conditions | 4 | 0,2 |  |  |  |
|  |  | 9 health-related conditions | 3 | 0,1 |  |  |  |
| **Bold:** statistically signficant; na = not applicable; # Includes living with partner, parents, children, roommates or others; ^ >100% as participants may have received multiple treatments | | | | | | | |
