# Supplementary material for: Health-related conditions among long-term cancer survivors diagnosed in adolescence and young adulthood (AYA): results of the SURVAYA study
Source: J Cancer Surviv. 2024 May 13;19(6):1821–34. doi: 10.1007/s11764-024-01597-0 (PMC12546281; doi:10.1007/s11764-024-01597-0)
Supplement: Supplementary file 3 — Supplementary file3 (DOCX 33 KB) [file 11764_2024_1597_MOESM3_ESM.docx]

**Appendix Table 3. Prevalence and latency time of health-related subconditions among AYA cancer survivors per health-related condition**

|  |  | **Post-cancer diagnosis** | | | |
| --- | --- | --- | --- | --- | --- |
|  |  | **N** | **%** | **Mean** | **SD** |
|  |  |  |  | **(months)** | |
| **Hearing conditions** | | 280 | 7.4 |  |  |
|  | **Hearing loss (hearing impairment requiring a hearing aid)** | 44 | 15,7 | 117,86 | 70,74 |
|  | **Deafness in one or both ears (not completely corrected by hearing aid)** | 29 | 10,4 | 101,24 | 58,36 |
|  | **Complete deafness in both ears** | 1 | 0,4 | 18,00 | . |
|  | **Tinnitus or ringing in the ears** | 174 | 62,1 | 87,86 | 61,10 |
|  | **Persistent dizziness or vertigo** | 26 | 9,3 | 93,27 | 66,09 |
|  | **Problems with hearing sounds, words or language in crowds** | 115 | 41,1 | 85,73 | 63,57 |
|  | **Any other hearing condition** | 61 | 21,8 | 88,20 | 57,60 |
| **Vision conditions** | | 568 | 15.0 |  |  |
|  | **Blind (in one or both eyes)** | 7 | 1,2 | 122,86 | 75,55 |
|  | **Cataract** | 71 | 12,5 | 98,63 | 65,29 |
|  | **Glaucoma (excess pressure in the eyeball)** | 20 | 3,5 | 123,95 | 53,62 |
|  | **Double vision** | 44 | 7,7 | 94,20 | 52,46 |
|  | **A detached retina or any other condition of the retina [open answer]** | 40 | 7,0 | 118,07 | 59,52 |
|  | **Any other trouble seeing with one or both eyes even when wearing glasses/ lenses (if you have them)** | 133 | 23,4 | 118,64 | 64,94 |
|  | **Very dry eyes requiring eye drops or ointment** | 201 | 35,4 | 98,28 | 63,66 |
|  | **Any other eye condition** | 285 | 50,2 | 110,85 | 58,41 |
| **Speech, taste and smell conditions** | | 170 | 4.5 |  |  |
|  | **Stammering or stuttering** | 23 | 13,5 | 61,48 | 54,09 |
|  | **Any other speech condition** | 49 | 28,8 | 74,02 | 58,86 |
|  | **Abnormal sense of taste** | 51 | 30,0 | 52,90 | 59,48 |
|  | **Loss of taste or smell for at least 3 months** | 103 | 60,6 | 68,11 | 61,39 |
| **Urinary tract conditions (bladder, kidneys)** | | 411 | 10.9 |  |  |
|  | **Kidney stones** | 66 | 16,1 | 101,77 | 59,45 |
|  | **Repeated kidney infections (so-called pyelonephritis)** | 14 | 3,4 | 85,36 | 61,93 |
|  | **Repeated bladder infections** | 211 | 51,3 | 85,68 | 64,25 |
|  | **Any other kind of kidney or urinary tract condition** | 193 | 47,0 | 91,56 | 61,07 |
| **Endocrine conditions** | | 531 | 14.1 |  |  |
|  | **An overactive thyroid gland (hyperthyroid)** | 45 | 8,5 | 82,76 | 60,51 |
|  | **An underactive thyroid gland (hypothyroid)** | 142 | 26,7 | 70,34 | 58,65 |
|  | **Thyroid nodule** | 27 | 5,1 | 86,48 | 69,33 |
|  | **Thyroid enlargement** | 15 | 2,8 | 103,93 | 69,14 |
|  | **Diabetes - controlled with diet** | 30 | 5,6 | 115,27 | 60,39 |
|  | **Diabetes - controlled with pills or tablets, but not with insulin shots** | 24 | 4,5 | 109,29 | 52,21 |
|  | **Diabetes - controlled with (amongst others) insulin shots** | 26 | 4,9 | 70,23 | 54,21 |
|  | **Deficiency of growth hormone** | 12 | 2,3 | 76,83 | 54,86 |
|  | **Osteoporosis, brittle, weak or fragile bones** | 218 | 41,1 | 78,08 | 59,13 |
|  | **Any other endocrine condition** | 122 | 23,0 | 59,84 | 51,67 |
| **Cardiovascular conditions** | | 443 | 11.7 |  |  |
|  | **Rheumatic heart disease** | 2 | 0,5 | 121,50 | 132,23 |
|  | **Hardening of the arteries or arteriosclerosis** | 28 | 6,3 | 133,43 | 69,03 |
|  | **Irregular heartbeat or palpitations (arrythmia) requiring medication or follow-up by a doctor** | 102 | 23,0 | 101,78 | 61,69 |
|  | **Congestive heart failure or cardiomyopathy (weak heart muscle)** | 39 | 8,8 | 91,41 | 72,25 |
|  | **Myocardial infarction (heart attack)** | 23 | 5,2 | 130,87 | 67,13 |
|  | **Narrowing or disease of the coronary artery** | 20 | 4,5 | 155,20 | 56,10 |
|  | **Hypertension (high blood pressure) not requiring medication** | 77 | 17,4 | 119,81 | 64,14 |
|  | **Hypertension (high blood pressure) requiring medication** | 182 | 41,1 | 100,76 | 59,80 |
|  | **A stroke such as a TIA (Transient Ischemic Attack), cerebrovascular accident (CVA), or hemorrhagic stroke** | 43 | 9,7 | 106,91 | 58,74 |
|  | **Angina pectoris (chest pains due to lack of oxygen to heart) requiring medication such as nitroglycerine** | 18 | 4,1 | 133,33 | 53,58 |
|  | **Pericarditis or fluid around the heart** | 14 | 3,2 | 88,00 | 55,68 |
|  | **Pericardial constriction (scarring or constricting of the sac around the heart)** | 2 | 0,5 | 53,00 | 7,07 |
|  | **Stiff or leaking heart valves** | 30 | 6,8 | 121,63 | 61,21 |
|  | **Blood clot in head, lung, arm, leg or pelvis (thrombosis or embolism)** | 33 | 7,4 | 87,58 | 60,64 |
|  | **Any other heart or circulatory problem** | 59 | 13,3 | 110,05 | 56,52 |
| **Respiratory conditions** | | 428 | 11.3 |  |  |
|  | **Bronchitis** | 47 | 11,0 | 81,98 | 55,59 |
|  | **Hay fever** | 130 | 30,4 | 72,65 | 57,11 |
|  | **Recurrent sinus infection** | 47 | 11,0 | 75,04 | 50,36 |
|  | **Tonsillitis or enlargement of the tonsils or adenoids** | 15 | 3,5 | 70,53 | 49,37 |
|  | **Pleurisy (inflammation of the lining of the lungs)** | 16 | 3,7 | 96,25 | 74,07 |
|  | **Asthma** | 60 | 14,0 | 85,98 | 65,22 |
|  | **Abnormal chest wall** | 4 | 0,9 | 95,50 | 51,99 |
|  | **Chronic cough or shortness of breath for more than one month** | 109 | 25,5 | 93,67 | 66,58 |
|  | **Pneuomania, 3 or more times in the past 2 years** | 33 | 7,7 | 123,12 | 67,78 |
|  | **Lungemphysema** | 13 | 3,0 | 130,23 | 56,90 |
|  | **Lung fibrosis or "scarring" of the lung** | 45 | 10,5 | 59,18 | 64,46 |
|  | **Any other breathing or lung problem** | 112 | 26,2 | 99,14 | 66,38 |
| **Digestive conditions** | | 568 | 15.0 |  |  |
|  | **Gallstones** | 95 | 16,7 | 103,00 | 60,56 |
|  | **Any other gallbladder condition** | 41 | 7,2 | 103,61 | 58,60 |
|  | **Cirrhosis of the liver** | 27 | 4,8 | 93,74 | 54,80 |
|  | **Hepatitis (inflammation of the liver, such as Hepatitis A, B, C, D, E)** | 6 | 1,1 | 77,00 | 61,94 |
|  | **Jaundice** | 4 | 0,7 | 71,75 | 81,89 |
|  | **Any other liver condition** | 35 | 6,2 | 89,46 | 64,94 |
|  | **A stomach ulcer** | 19 | 3,3 | 83,53 | 54,11 |
|  | **A condition of the esophagus** | 38 | 6,7 | 83,08 | 59,83 |
|  | **Frequent stomach pain (dyspepsia)** | 66 | 11,6 | 95,61 | 59,33 |
|  | **Frequent heartburn** | 152 | 26,8 | 85,12 | 61,67 |
|  | **Any other stomach condition** | 77 | 13,6 | 105,61 | 67,10 |
|  | **Intestinal polyps** | 50 | 8,8 | 128,70 | 60,72 |
|  | **Diverticular disease** | 31 | 5,5 | 107,90 | 74,21 |
|  | **Colitis (such as Chrohn's disease or ulcerative colitis)** | 16 | 2,8 | 107,81 | 68,50 |
|  | **Frequent constipation** | 120 | 21,1 | 92,14 | 67,02 |
|  | **Chronic diarrhea (more than 3 weeks ánd more than 3 days per week ánd more than 3 times per day)** | 81 | 14,3 | 82,78 | 65,22 |
|  | **Rectal or anal fistula** | 17 | 3,0 | 66,06 | 56,22 |
|  | **Rectal or anal stricture (narrowing or scarring)** | 17 | 3,0 | 72,06 | 68,88 |
| **Rheumatoid arthritis** | | 79 | 2.1 | 98,73 | 63,03 |
| **Arthrosis** | | 261 | 6.9 | 115,31 | 62,14 |
| **Depression** | | 325 | 8.6 | 78,66 | 58,12 |
| **Secondary malignancy (no recurrence or metastasis)** | | 241 | 6.4 | 105,46 | 64,61 |
| FOOTNOTE: Latency time in months; the cut-off for categorizing health-related conditions as diagnosed after the cancer diagnosis (i.e. 6 months) was chosen based on clinical input, as no golden standard for this limit was available. SD: standard deviation. | | | | | |
