# Supplementary material for: Health-related conditions among long-term cancer survivors diagnosed in adolescence and young adulthood (AYA): results of the SURVAYA study
Source: J Cancer Surviv. 2024 May 13;19(6):1821–34. doi: 10.1007/s11764-024-01597-0 (PMC12546281; doi:10.1007/s11764-024-01597-0)
Supplement: Supplementary file 4 — Supplementary file4 (DOCX 87 KB) [file 11764_2024_1597_MOESM4_ESM.docx]

**Appendix Table 4a-l. Associations between sociodemographic and clinical variables and health-related conditions among AYA cancer survivors (per health-related condition)**

**Appendix Table 4a.**

|  |  |  | **Hearing condition (N= 3776)** | | | | | |
| --- | --- | --- | --- | --- | --- | --- | --- | --- |
|  |  |  | **Univariable logistic regression** | | | **Multivariable logistic regression** | | |
|  |  |  |  | | | **Nagelkerkes R^2^= 0,041** | | |
|  |  |  | **OR** | **95% CI** | **p-value** | **OR** | **95% CI** | **p-value** |
| Age at diagnosis in years |  | 18-24 years | REF |  |  | REF |  |  |
|  |  | 25-34 years | 0,679 | 0,481-0,958 | 0,027 | 0,765 | 0,515-1,138 | 0,186 |
|  |  | 35-39 years | 0,872 | 0,622-1,222 | 0,426 | 0,978 | 0,648-1,475 | 0,914 |
| Time since diagnosis in years |  | <11 years | REF |  |  | REF |  |  |
|  |  | 11-15 years | 1,593 | 1,186-2,140 | 0,002 | 1,603 | 1,170-2,196 | 0,003 |
|  |  | >15 years | 1,754 | 1,283-2,397 | <0,001 | 1,525 | 1,082-2,149 | 0,016 |
| Sex |  | Male | REF |  |  | REF |  |  |
|  |  | Female | 0,643 | 0,504-0,821 | <0,001 | 0,593 | 0,379-0,928 | 0,022 |
| Partner status at time of questionnaire |  | Partner | 0,941 | 0,681-1,301 | 0,712 |  |  |  |
|  |  | No partner | REF |  |  |  |  |  |
| Education (achieved) |  | No education or primary education | 0,561 | 0,074-4,225 | 0,575 | 0,570 | 0,074-4,408 | 0,591 |
|  |  | Secondary education | 1,541 | 0,956-2,485 | 0,076 | 1,307 | 0,769-2,222 | 0,323 |
|  |  | Secondary vocational education | 1,037 | 0,745-1,444 | 0,830 | 1,011 | 0,707-1,445 | 0,952 |
|  |  | Higher (vocational) education | 0,959 | 0,683-1,347 | 0,810 | 0,959 | 0,667-1,378 | 0,821 |
|  |  | University education | REF |  |  | REF |  |  |
| Living status |  | Living alone | 1,191 | 0,839-1,689 | 0,328 |  |  |  |
|  |  | Not living alone# | REF |  |  |  |  |  |
| Tumor type |  | Breast | REF |  |  | REF |  |  |
|  |  | Digestive track, other | 0,454 | 0,061-3,386 | 0,441 | 0,280 | 0,036-2,180 | 0,224 |
|  |  | Colon and rectal | 0,541 | 0,166-1,765 | 0,308 | 0,334 | 0,097-1,153 | 0,083 |
|  |  | Bone and soft tissue | 0,666 | 0,313-1,418 | 0,292 | 0,483 | 0,203-1,148 | 0,099 |
|  |  | Respiratory tract | 1,053 | 0,244-4,546 | 0,945 | 0,925 | 0,202-4,239 | 0,920 |
|  |  | Melanoma | 0,902 | 0,518-1,570 | 0,715 | 0,718 | 0,389-1,324 | 0,289 |
|  |  | Other | 0,000 | 0,000 . | 0,999 | 0,000 | 0,000 . | 0,999 |
|  |  | Germ cell | 1,586 | 1,112-2,263 | 0,011 | 0,909 | 0,498-1,658 | 0,756 |
|  |  | Head and neck | 2,713 | 1,573-4,680 | <0,001 | 1,721 | 0,884-3,353 | 0,110 |
|  |  | Female genitalia | 0,820 | 0,505-1,333 | 0,424 | 0,858 | 0,515-1,428 | 0,555 |
|  |  | Male genitalia | 0,000 | 0,000 . | 0,999 | 0,000 | 0,000 . | 0,999 |
|  |  | Urinary tract | 1,039 | 0,312-3,459 | 0,950 | 0,843 | 0,243-2,919 | 0,787 |
|  |  | Lymphoid hematological | 0,902 | 0,591-1,379 | 0,635 | 0,604 | 0,338-1,080 | 0,089 |
|  |  | Myeloid hematological | 1,012 | 0,507-2,023 | 0,973 | , | , | , |
|  |  | Thyroid gland | 0,715 | 0,379-1,348 | 0,299 | 0,668 | 0,337-1,327 | 0,249 |
|  |  | Central nervous system | 0,982 | 0,492-1,961 | 0,959 | , | , | , |
| Primary treatment | Chemotherapy | No | REF |  |  |  |  |  |
|  |  | Yes | 1,181 | 0,921-1,513 | 0,190 |  |  |  |
|  | Radiotherapy | No | REF |  |  |  |  |  |
|  |  | Yes | 1,196 | 0,938-1,526 | 0,149 |  |  |  |
|  | Endocrine therapy | No | REF |  |  |  |  |  |
|  |  | Yes | 1,031 | 0,713-1,490 | 0,871 |  |  |  |
|  | Targeted therapy | No | REF |  |  |  |  |  |
|  |  | Yes | 0,660 | 0,386-1,126 | 0,127 |  |  |  |
|  | Surgery | No | REF |  |  |  |  |  |
|  |  | Yes | 0,944 | 0,706-1,261 | 0,695 |  |  |  |
|  | Stem cell therapy | No | REF |  |  |  |  |  |
|  |  | Yes | 1,334 | 0,743-2,394 | 0,335 |  |  |  |
| Tumor stage |  | I | REF |  |  | REF |  |  |
|  |  | II | 0,986 | 0,722-1,349 | 0,932 | 0,990 | 0,705-1,390 | 0,954 |
|  |  | III | 1,447 | 1,025-2,043 | 0,036 | 1,347 | 0,931-1,948 | 0,114 |
|  |  | IV | 1,477 | 0,864-2,525 | 0,154 | 1,543 | 0,841-2,833 | 0,161 |
| No multicollinearity was found for any of the independent variables [multicollinearity is indicated by: tolerance value <0.1, variance inflation factor value > 10 and variance proportions ≥0.7 on the same eigenvalue]; * Should be interpreted with caution due to wide 95% confidence intervals; REF: reference group; OR: odds ratio; CI: confidence interval; # Includes living with partner, parents, children, roommates or others. | | | | | | | | |

**Appendix Table 4b.**

|  |  |  | **Vision conditions (N= 3776)** | | | | | |
| --- | --- | --- | --- | --- | --- | --- | --- | --- |
|  |  |  | **Univariable logistic regression** | | | **Multivariable logistic regression** | | |
|  |  |  |  | | | **Nagelkerkes R^2^= 0,071** | | |
|  |  |  | **OR** | **95% CI** | **p-value** | **OR** | **95% CI** | **p-value** |
| Age at diagnosis in years |  | 18-24 years | REF |  |  | REF |  |  |
|  |  | 25-34 years | 0,934 | 0,708-1,233 | 0,629 | 0,933 | 0,665-1,309 | 0,687 |
|  |  | 35-39 years | 1,426 | 1,088-1,869 | 0,010 | 1,39 | 0,981-1,970 | 0,064 |
| Time since diagnosis in years |  | <11 years | REF |  |  | REF |  |  |
|  |  | 11-15 years | 1,685 | 1,356-2,094 | <0,001 | 2,109 | 1,645-2,704 | <0,001 |
|  |  | >15 years | 1,983 | 1,578-2,493 | <0,001 | 2,336 | 1,789-3,049 | <0,001 |
| Sex |  | Male | REF |  |  | REF |  |  |
|  |  | Female | 1,477 | 1,221-1,788 | <0,001 | 1,112 | 0,802-1,540 | 0,525 |
| Partner status at time of questionnaire |  | Partner | 0,811 | 0,644-1,020 | 0,074 | 1,003 | 0,698-1,441 | 0,989 |
|  |  | No partner | REF |  |  | REF |  |  |
| Education (achieved) |  | No education or primary education | 1,359 | 0,456-4,052 | ,582 | 1,283 | 0,356-4,622 | 0,703 |
|  |  | Secondary education | 1,430 | 0,975-2,098 | ,067 | 0,949 | 0,594-1,517 | 0,827 |
|  |  | Secondary vocational education | 1,370 | 1,069-1,754 | ,013 | 1,255 | 0,949-1,661 | 0,111 |
|  |  | Higher (vocational) education | 1,127 | 0,872-1,457 | ,360 | 1,102 | 0,828-1,468 | 0,506 |
|  |  | University education | REF |  |  | REF |  |  |
| Living status |  | Living alone | 1,265 | 0,981-1,633 | 0,070 | 1,085 | 0,724-1,627 | 0,692 |
|  |  | Not living alone# | REF |  |  | REF |  |  |
| Tumor type |  | Breast | REF |  |  | REF |  |  |
|  |  | Digestive track, other | 0,000 | 0,000 . | 0,998 | 0 | 0,000 . | 0,998 |
|  |  | Colon and rectal | 0,634 | 0,309-1,298 | 0,212 | 0,816 | 0,370-1,801 | 0,615 |
|  |  | Bone and soft tissue | 0,721 | 0,446-1,166 | 0,182 | 0,812 | 0,433-1,523 | 0,516 |
|  |  | Respiratory tract | 0,590 | 0,175-1,983 | 0,393 | 0,598 | 0,127-2,810 | 0,515 |
|  |  | Melanoma | 0,557 | 0,363-0,855 | 0,007 | 0,913 | 0,510-1,632 | 0,758 |
|  |  | Other | 2,022 | 0,517-7,906 | 0,311 | 0 | 0,000 . | 0,999 |
|  |  | Germ cell | 0,395 | 0,283-0,552 | <0,001 | 0,609 | 0,356-1,041 | 0,070 |
|  |  | Head and neck | 0,973 | 0,583-1,622 | 0,916 | 1,124 | 0,565-2,237 | 0,739 |
|  |  | Female genitalia | 0,811 | 0,587-1,121 | 0,205 | 1,024 | 0,653-1,606 | 0,916 |
|  |  | Male genitalia | 0,000 | 0,000 . | 0,999 | 0 | 0,000 . | 0,999 |
|  |  | Urinary tract | 0,971 | 0,423-2,231 | 0,945 | 1,574 | 0,626-3,959 | 0,335 |
|  |  | Lymphoid hematological | 1,112 | 0,847-1,460 | 0,445 | 0,781 | 0,399-1,532 | 0,473 |
|  |  | Myeloid hematological | 2,162 | 1,455-3,214 | <0,001 | , | , | , |
|  |  | Thyroid gland | 0,862 | 0,581-1,280 | 0,462 | 1,4 | 0,834-2,350 | 0,202 |
|  |  | Central nervous system | 0,851 | 0,523-1,383 | 0,514 | , | , | , |
| Primary treatment | Chemotherapy | No | REF |  |  | REF |  |  |
|  |  | Yes | 1,412 | 1,174-1,698 | <0,001 | 1,363 | 0,977-1,902 | 0,068 |
|  | Radiotherapy | No | REF |  |  | REF |  |  |
|  |  | Yes | 1,240 | 1,037-1,483 | 0,018 | 1,06 | 0,825-1,363 | 0,648 |
|  | Endocrine therapy | No | REF |  |  | REF |  |  |
|  |  | Yes | 1,422 | 1,107-1,828 | 0,006 | 1,222 | 0,855-1,746 | 0,272 |
|  | Targeted therapy | No | REF |  |  |  |  |  |
|  |  | Yes | 1,097 | 0,792-1,519 | 0,578 |  |  |  |
|  | Surgery | No | REF |  |  | REF |  |  |
|  |  | Yes | 0,566 | 0,465-0,690 | <0,001 | 0,634 | 0,365-1,102 | 0,106 |
|  | Stem cell therapy | No | REF |  |  | REF |  |  |
|  |  | Yes | 3,752 | 2,622-5,367 | <0,001 | 1,015 | 0,419-2,458 | 0,974 |
| Tumor stage |  | I | REF |  |  | REF |  |  |
|  |  | II | 1,261 | 1,007-1,579 | 0,043 | 1,046 | 0,796-1,374 | 0,747 |
|  |  | III | 0,938 | 0,697-1,263 | 0,673 | 0,93 | 0,654-1,321 | 0,683 |
|  |  | IV | 1,552 | 1,029-2,341 | 0,036 | 1,267 | 0,776-2,067 | 0,344 |
| No multicollinearity was found for any of the independent variables [multicollinearity is indicated by: tolerance value <0.1, variance inflation factor value > 10 and variance proportions ≥0.7 on the same eigenvalue]; * Should be interpreted with caution due to wide 95% confidence intervals; REF: reference group; OR: odds ratio; CI: confidence interval; # Includes living with partner, parents, children, roommates or others. | | | | | | | | |

**Appendix Table 4c.**

|  |  |  | **Speech, taste & smell conditions (N= 3776)** | | | | | |
| --- | --- | --- | --- | --- | --- | --- | --- | --- |
|  |  |  | **Univariable logistic regression** | | | **Multivariable logistic regression** | | |
|  |  |  |  | | | **Nagelkerkes R^2^= 0,068** | | |
|  |  |  | **OR** | **95% CI** | **p-value** | **OR** | **95% CI** | **p-value** |
| Age at diagnosis in years |  | 18-24 years | REF |  |  |  |  |  |
|  |  | 25-34 years | 0,840 | 0,543-1,300 | 0,433 |  |  |  |
|  |  | 35-39 years | 0,863 | 0,555-1,340 | 0,511 |  |  |  |
| Time since diagnosis in years |  | <11 years | REF |  |  |  |  |  |
|  |  | 11-15 years | 0,757 | 0,525-1,090 | 0,134 |  |  |  |
|  |  | >15 years | 0,,927 | 0,634-1,356 | 0,697 |  |  |  |
| Sex |  | Male | REF |  |  |  |  |  |
|  |  | Female | 0,930 | 0,680-1,272 | 0,650 |  |  |  |
| Partner status at time of questionnaire |  | Partner | 0,696 | 0,479-1,013 | 0,058 | 0,752 | 0,484 1,170 | 0,206 |
|  |  | No partner | REF |  |  | REF |  |  |
| Education (achieved) |  | No education or primary education | 1,207 | 0,158-9,246 | 0,856 | 1,328 | 0,159 11,087 | 0,793* |
|  |  | Secondary education | 2,000 | 1,080-3,702 | 0,027 | 1,02 | 0,451 2,306 | 0,963 |
|  |  | Secondary vocational education | 1,426 | 0,914-2,224 | 0,117 | 1,284 | 0,798 2,068 | 0,303 |
|  |  | Higher (vocational) education | 1,264 | 0,800-1,996 | 0,315 | 1,104 | 0,676 1,803 | 0,693 |
|  |  | University education | REF |  |  | REF |  |  |
| Living status |  | Living alone | 1,345 | 0,881-2,053 | 0,170 |  |  |  |
|  |  | Not living alone# | REF |  |  |  |  |  |
| Tumor type |  | Breast | REF |  |  | REF |  |  |
|  |  | Digestive track, other | 0,698 | 0,093-5,247 | 0,727 | 0,739 | 0,096 5,712 | 0,772 |
|  |  | Colon and rectal | 1,425 | 0,547-3,716 | 0,469 | 1,443 | 0,533 3,911 | 0,471 |
|  |  | Bone and soft tissue | 0,372 | 0,114-1,216 | 0,102 | 0,407 | 0,123 1,344 | 0,140 |
|  |  | Respiratory tract | 1,619 | 0,371-7,064 | 0,521 | 0,81 | 0,100 6,564 | 0,844 |
|  |  | Melanoma | 0,232 | 0,071-0,754 | 0,015 | 0,271 | 0,079 ,929 | 0,038 |
|  |  | Other | 2,249 | 0,278-18,164 | 0,447 | 0 | 0,000 . | 0,999 |
|  |  | Germ cell | 0,600 | 0,346-1,041 | 0,069 | 0,706 | 0,392 1,272 | 0,246 |
|  |  | Head and neck | 3,924 | 2,195-7,014 | <0,001 | 3,254 | 1,677 6,315 | <,001 |
|  |  | Female genitalia | 0,455 | 0,220-0,945 | 0,035 | 0,411 | 0,184 ,917 | 0,030 |
|  |  | Male genitalia | 0,000 | 0,000 . | 0,999 | 0 | ,000 . | 0,999 |
|  |  | Urinary tract | 0,000 | 0,000 . | 0,998 | 0 | ,000 . | 0,998 |
|  |  | Lymphoid hematological | 1,306 | 0,820-2,079 | 0,261 | 0,688 | 0,270 1,756 | 0,434 |
|  |  | Myeloid hematological | 1,390 | 0,661-2,923 | 0,385 | , | , | , |
|  |  | Thyroid gland | 0,813 | 0,390-1,695 | 0,581 | 0,703 | 0,325 1,519 | 0,370 |
|  |  | Central nervous system | 2,179 | 1,158-4,102 | 0,016 | , | , | , |
| Primary treatment | Chemotherapy | No | REF |  |  |  |  |  |
|  |  | Yes | 1,175 | 0,858-1,608 | 0,314 |  |  |  |
|  | Radiotherapy | No | REF |  |  | REF |  |  |
|  |  | Yes | 1,604 | 1,173-2,192 | 0,003 | 1,242 | 0,825 1,871 | 0,300 |
|  | Endocrine therapy | No | REF |  |  |  |  |  |
|  |  | Yes | 1,015 | 0,636-1,621 | 0,949 |  |  |  |
|  | Targeted therapy | No | REF |  |  |  |  |  |
|  |  | Yes | 1,257 | 0,740-2,134 | 0,397 |  |  |  |
|  | Surgery | No | REF |  |  | REF |  |  |
|  |  | Yes | 0,570 | 0,410-0,794 | <0,001 | 0,623 | 0,280 1,384 | 0,245 |
|  | Stem cell therapy | No | REF |  |  | REF |  |  |
|  |  | Yes | 2,784 | 1,590-4,875 | <0,001 | 2,008 | 0,645 6,255 | 0,229 |
| Tumor stage |  | I | REF |  |  | REF |  |  |
|  |  | II | 1,154 | 0,771-1,728 | 0,487 | 0,866 | 0,557 1,346 | 0,523 |
|  |  | III | 0,964 | 0,569-1,631 | 0,890 | 0,702 | 0,396 1,243 | 0,225 |
|  |  | IV | 3,254 | 1,891-5,600 | <0,001 | 1,64 | 0,848 3,170 | 0,141 |
| No multicollinearity was found for any of the independent variables [multicollinearity is indicated by: tolerance value <0.1, variance inflation factor value > 10 and variance proportions ≥0.7 on the same eigenvalue]; * Should be interpreted with caution due to wide 95% confidence intervals; REF: reference group; OR: odds ratio; CI: confidence interval; # Includes living with partner, parents, children, roommates or others. | | | | | | | | |

**Appendix Table 4d.**

|  |  |  | **Urinary tract conditions (N= 3776)** | | | | | |
| --- | --- | --- | --- | --- | --- | --- | --- | --- |
|  |  |  | **Univariable logistic regression** | | | **Multivariable logistic regression** | | |
|  |  |  |  | | | **Nagelkerkes R^2^= 0,071** | | |
|  |  |  | **OR** | **95% CI** | **p-value** | **OR** | **95% CI** | **p-value** |
| Age at diagnosis in years |  | 18-24 years | REF |  |  |  |  |  |
|  |  | 25-34 years | 1,086 | 0,792-1,488 | 0,61 |  |  |  |
|  |  | 35-39 years | 1,238 | 0,904-1,696 | 0,18 |  |  |  |
| Time since diagnosis in years |  | <11 years | REF |  |  | REF |  |  |
|  |  | 11-15 years | 1,263 | 0,985-1,619 | 0,07 | 1,441 | 1,097-1,892 | 0,01 |
|  |  | >15 years | 1,722 | 1,336-2,221 | <0,001 | 1,992 | 1,498-2,649 | <0,001 |
| Sex |  | Male | REF |  |  | REF |  |  |
|  |  | Female | 1,583 | 1,267-1,978 | <0,001 | 1,238 | 0,836-1,834 | 0,29 |
| Partner status at time of questionnaire |  | Partner | 0,795 | 0,612-1,031 | 0,08 | 0,733 | 0,550-0,975 | 0,03 |
|  |  | No partner | REF |  |  | REF |  |  |
| Education (achieved) |  | No education or primary education | 1,310 | 0,383-4,487 | 0,67 |  |  |  |
|  |  | Secondary education | 1,187 | 0,758-1,860 | 0,45 |  |  |  |
|  |  | Secondary vocational education | 1,229 | 0,928-1,627 | 0,15 |  |  |  |
|  |  | Higher (vocational) education | 1,076 | 0,805-1,437 | 0,62 |  |  |  |
|  |  | University education | REF |  |  |  |  |  |
| Living status |  | Living alone | 1,044 | 0,768-1,418 | 0,78 |  |  |  |
|  |  | Not living alone# | REF |  |  |  |  |  |
| Tumor type |  | Breast | REF |  |  | REF |  |  |
|  |  | Digestive track, other | 0,678 | 0,159-2,896 | 0,60 | 0,75 | 0,169-3,326 | 0,71 |
|  |  | Colon and rectal | 1,780 | 0,924-3,430 | 0,09 | 2,038 | 0,995-4,176 | 0,05 |
|  |  | Bone and soft tissue | 0,609 | 0,309-1,198 | 0,15 | 0,566 | 0,261-1,229 | 0,15 |
|  |  | Respiratory tract | 1,187 | 0,350-4,023 | 0,78 | 0,999 | 0,226-4,411 | 1,00 |
|  |  | Melanoma | 0,651 | 0,379-1,117 | 0,12 | 0,682 | 0,388-1,199 | 0,18 |
|  |  | Other | 2,374 | 0,496-11,358 | 0,28 | 7,209 | 0,636-81,684 | 0,11* |
|  |  | Germ cell | 0,829 | 0,579-1,187 | 0,31 | 1,017 | 0,590-1,751 | 0,95 |
|  |  | Head and neck | 0,513 | 0,219-1,202 | 0,13 | 0,539 | 0,219-1,326 | 0,18 |
|  |  | Female genitalia | 2,833 | 2,055-3,905 | <0,001 | 2,818 | 1,977-4,016 | <0,001 |
|  |  | Male genitalia | 0,000 | 0,000 . | 0,999 | 0 | 0,000 . | 1,00 |
|  |  | Urinary tract | 2,302 | 1,030-5,143 | 0,04 | 2,668 | 1,153-6,176 | 0,02 |
|  |  | Lymphoid hematological | 0,991 | 0,691-1,421 | 0,96 | 0,819 | 0,515-1,305 | 0,40 |
|  |  | Myeloid hematological | 1,225 | 0,695-2,159 | 0,48 | , | , | , |
|  |  | Thyroid gland | 1,678 | 1,099-2,562 | 0,02 | 1,851 | 1,158-2,961 | 0,01 |
|  |  | Central nervous system | 1,104 | 0,618-1,971 | 0,74 | , | , | , |
| Primary treatment | Chemotherapy | No | REF |  |  |  |  |  |
|  |  | Yes | 0,965 | 0,785-1,186 | 0,76 |  |  |  |
|  | Radiotherapy | No | REF |  |  |  |  |  |
|  |  | Yes | 1,181 | 0,962-1,449 | 0,11 |  |  |  |
|  | Endocrine therapy | No | REF |  |  |  |  |  |
|  |  | Yes | 0,897 | 0,649-1,240 | 0,51 |  |  |  |
|  | Targeted therapy | No | REF |  |  | REF |  |  |
|  |  | Yes | 0,616 | 0,391-0,973 | 0,04 | 0,881 | 0,517-1,502 | 0,64 |
|  | Surgery | No | REF |  |  |  |  |  |
|  |  | Yes | 0,871 | 0,685-1,108 | 0,26 |  |  |  |
|  | Stem cell therapy | No | REF |  |  | REF |  |  |
|  |  | Yes | 1,611 | 1,008-2,573 | 0,05 | 2,376 | 0,971-5,817 | 0,06 |
| Tumor stage |  | I | REF |  |  | REF |  |  |
|  |  | II | 0,754 | 0,581-0,978 | 0,03 | 1,019 | 0,760-1,368 | 0,90 |
|  |  | III | 0,778 | 0,563-1,074 | 0,13 | 1,05 | 0,733-1,503 | 0,79 |
|  |  | IV | 1,061 | 0,662-1,700 | 0,81 | 1,482 | 0,859-2,556 | 0,16 |
| No multicollinearity was found for any of the independent variables [multicollinearity is indicated by: tolerance value <0.1, variance inflation factor value > 10 and variance proportions ≥0.7 on the same eigenvalue]; * Should be interpreted with caution due to wide 95% confidence intervals; REF: reference group; OR: odds ratio; CI: confidence interval; # Includes living with partner, parents, children, roommates or others. | | | | | | | | |

**Appendix Table 4e.**

|  |  |  | **Endocrine conditions (N= 3776)** | | | | | |
| --- | --- | --- | --- | --- | --- | --- | --- | --- |
|  |  |  | **Univariable logistic regression** | | | **Multivariable logistic regression** | | |
|  |  |  |  | | | **Nagelkerkes R^2^= 0,107** | | |
|  |  |  | **OR** | **95% CI** | **p-value** | **OR** | **95% CI** | **p-value** |
| Age at diagnosis in years |  | 18-24 years | REF |  |  | REF |  |  |
|  |  | 25-34 years | 1,006 | 0,757-1,338 | 0,97 | 1,197 | 0,841-1,705 | 0,32 |
|  |  | 35-39 years | 1,374 | 1,038-1,818 | 0,03 | 1,449 | 1,001-2,098 | 0,05 |
| Time since diagnosis in years |  | <11 years | REF |  |  | REF |  |  |
|  |  | 11-15 years | 1,206 | 0,968-1,503 | 0,09 | 1,294 | 1,013-1,654 | 0,04 |
|  |  | >15 years | 1,553 | 1,236-1,952 | <0,001 | 1,671 | 1,291-2,163 | <0,001 |
| Sex |  | Male | REF |  |  | REF |  |  |
|  |  | Female | 2,354 | 1,902-2,914 | <0,001 | 2,834 | 1,959-4,099 | <0,001 |
| Partner status at time of questionnaire |  | Partner | 1,008 | 0,787-1,291 | 0,95 |  |  |  |
|  |  | No partner | REF |  |  |  |  |  |
| Education (achieved) |  | No education or primary education | 0,327 | 0,044-2,445 | 0,28 | 0,505 | 0,065-3,907 | 0,51 |
|  |  | Secondary education | 1,758 | 1,204-2,567 | 0,003 | 1,523 | 0,973-2,383 | 0,07 |
|  |  | Secondary vocational education | 1,290 | 0,996-1,671 | 0,05 | 1,352 | 1,011-1,809 | 0,04 |
|  |  | Higher (vocational) education | 1,246 | 0,958-1,620 | 0,10 | 1,303 | 0,973-1,745 | 0,08 |
|  |  | University education | REF |  |  | REF |  |  |
| Living status |  | Living alone | 0,938 | 0,707-1,245 | 0,66 |  |  |  |
|  |  | Not living alone# | REF |  |  |  |  |  |
| Tumor type |  | Breast | REF |  |  | REF |  |  |
|  |  | Digestive track, other | 0,924 | 0,372-2,292 | 0,86 | 2,726 | 0,988-7,519 | 0,05 |
|  |  | Colon and rectal | 0,317 | 0,135-0,740 | 0,008 | 0,715 | 0,289-1,770 | 0,47 |
|  |  | Bone and soft tissue | 0,340 | 0,192-0,602 | <0,001 | 0,597 | 0,289-1,237 | 0,17 |
|  |  | Respiratory tract | 0,296 | 0,069-1,259 | 0,099 | 0,358 | 0,045-2,836 | 0,33 |
|  |  | Melanoma | 0,222 | 0,129-0,382 | <0,001 | 0,521 | 0,267-1,016 | 0,06 |
|  |  | Other | 0,924 | 0,195-4,386 | 0,92 | 0 | 0,000 . | 1,00 |
|  |  | Germ cell | 0,296 | 0,213-0,413 | <0,001 | 1,324 | 0,752-2,331 | 0,33 |
|  |  | Head and neck | 0,628 | 0,367-01,076 | 0,09 | 1,248 | 0,618-2,520 | 0,54 |
|  |  | Female genitalia | 0,457 | 0,322-0,647 | <0,001 | 0,681 | 0,425-1,091 | 0,11 |
|  |  | Male genitalia | 0,000 | 0,000 . | 0,999 | 0 | 0,000 . | 1,00 |
|  |  | Urinary tract | 0,399 | 0,141-1,135 | 0,09 | 1,032 | 0,337-3,162 | 0,96 |
|  |  | Lymphoid hematological | 0,984 | 0,760-1,275 | 0,90 | 1,236 | 0,630-2,422 | 0,54 |
|  |  | Myeloid hematological | 1,008 | 0,653-1,556 | 0,97 | , | , | , |
|  |  | Thyroid gland | 0,424 | 0,270-0,667 | <0,001 | 0,912 | 0,522-1,596 | 0,75 |
|  |  | Central nervous system | 0,246 | 0,123-0,493 | <0,001 | , | , | , |
| Primary treatment | Chemotherapy | No | REF |  |  | REF |  |  |
|  |  | Yes | 2,159 | 1,766-2,639 | <0,001 | 1,089 | 0,772-1,535 | 0,63 |
|  | Radiotherapy | No | REF |  |  | REF |  |  |
|  |  | Yes | 1,648 | 1,368-1,985 | <0,001 | 1,126 | 0,877-1,444 | 0,35 |
|  | Endocrine therapy | No | REF |  |  | REF |  |  |
|  |  | Yes | 2,463 | 1,952-3,109 | <0,001 | 1,691 | 1,210-2,362 | 0,002 |
|  | Targeted therapy | No | REF |  |  |  |  |  |
|  |  | Yes | 1,130 | 0 ,811-1,575 | 0,47 |  |  |  |
|  | Surgery | No | REF |  |  | REF |  |  |
|  |  | Yes | 0,527 | 0,431-0,644 | <0,001 | 0,543 | 0,307-0,962 | 0,04 |
|  | Stem cell therapy | No | REF |  |  | REF |  |  |
|  |  | Yes | 2,777 | 1,902-4,055 | <0,001 | 1,216 | 0,499-2,963 | 0,67 |
| Tumor stage |  | I | REF |  |  | REF |  |  |
|  |  | II | 1,850 | 1,476-2,318 | <0,001 | 1,277 | 0,969-1,682 | 0,08 |
|  |  | III | 1,398 | 1,048-1,866 | 0,02 | 1,262 | 0,892-1,784 | 0,19 |
|  |  | IV | 1,484 | 0,950-2,318 | 0,08 | 1,049 | 0,620-1,774 | 0,86 |
| No multicollinearity was found for any of the independent variables [multicollinearity is indicated by: tolerance value <0.1, variance inflation factor value > 10 and variance proportions ≥0.7 on the same eigenvalue]; * Should be interpreted with caution due to wide 95% confidence intervals; REF: reference group; OR: odds ratio; CI: confidence interval; # Includes living with partner, parents, children, roommates or others. | | | | | | | | |

**Appendix Table 4f.**

|  |  |  | **Cardiovascular conditions (N= 3776)** | | | | | |
| --- | --- | --- | --- | --- | --- | --- | --- | --- |
|  |  |  | **Univariable logistic regression** | | | **Multivariable logistic regression** | | |
|  |  |  |  | | | **Nagelkerkes R^2^= 0,091** | | |
|  |  |  | **OR** | **95% CI** | **p-value** | **OR** | **95% CI** | **p-value** |
| Age at diagnosis in years |  | 18-24 years | REF |  |  | REF |  |  |
|  |  | 25-34 years | 1,045 | 0,765-1,427 | 0,78 | 1,276 | 0,885-1,841 | 0,19 |
|  |  | 35-39 years | 1,422 | 1,047-1,931 | 0,02 | 1,926 | 1,317-2,817 | <0,001 |
| Time since diagnosis in years |  | <11 years | REF |  |  | REF |  |  |
|  |  | 11-15 years | 1,845 | 1,427-2,385 | <0,001 | 1,843 | 1,388-2,446 | <0,001 |
|  |  | >15 years | 3,019 | 2,337-3,899 | <0,001 | 2,712 | 2,032-3,620 | <0,001 |
| Sex |  | Male | REF |  |  |  |  |  |
|  |  | Female | 0,884 | 0,723-1,082 | 0,23 |  |  |  |
| Partner status at time of questionnaire |  | Partner | 0,752 | 0,586-0,967 | 0,026 | 1,068 | 0,719-1,586 | 0,75 |
|  |  | No partner | REF |  |  | REF |  |  |
| Education (achieved) |  | No education or primary education | 0,551 | 0,073-4,149 | 0,56 | 0,744 | 0,093-5,954 | 0,78 |
|  |  | Secondary education | 2,035 | 1,308-3,165 | 0,002 | 1,845 | 1,119-3,043 | 0,02 |
|  |  | Secondary vocational education | 1,903 | 1,404-2,580 | <0,001 | 1,886 | 1,339-2,656 | <0,001 |
|  |  | Higher (vocational) education | 1,865 | 1,371-2,537 | <0,001 | 1,907 | 1,352-2,691 | <0,001 |
|  |  | University education | REF |  |  | REF |  |  |
| Living status |  | Living alone | 1,669 | 1,281-2,174 | <0,001 | 1,755 | 1,159-2,659 | 0,01 |
|  |  | Not living alone# | REF |  |  |  |  |  |
| Tumor type |  | Breast | REF |  |  | REF |  |  |
|  |  | Digestive track, other | 1,354 | 0,462-3,967 | 0,58 | 1,246 | 0,353-4,393 | 0,73 |
|  |  | Colon and rectal | 1,036 | 0,482-2,223 | 0,93 | 1,144 | 0,514-2,547 | 0,74 |
|  |  | Bone and soft tissue | 1,206 | 0,720-2,018 | 0,48 | 1,627 | 0,889-2,980 | 0,12 |
|  |  | Respiratory tract | 0,000 | 0,000 . | 0,998 | 0 | ,000 . | 1,00 |
|  |  | Melanoma | 0,641 | 0,379-1,085 | 0,098 | 1,129 | 0,583-2,185 | 0,72 |
|  |  | Other | 0,978 | 0,123-7,808 | 0,98 | 13,22 | 1,058-165,120 | 0,05* |
|  |  | Germ cell | 1,028 | 0,738-1,430 | 0,87 | 1,51 | 1,014-2,249 | 0,04 |
|  |  | Head and neck | 1,006 | 0,533-1,899 | 0,99 | 1,204 | 0,571-2,538 | 0,63 |
|  |  | Female genitalia | 0,981 | 0,665-1,447 | 0,92 | 1,275 | 0,774-2,101 | 0,34 |
|  |  | Male genitalia | 5,868 | 0,968-35,581 | 0,05 | 7,765 | 1,172-51,448 | 0,03* |
|  |  | Urinary tract | 0,952 | 0,332-2,731 | 0,93 | 1,686 | 0,549-5,176 | 0,36 |
|  |  | Lymphoid hematological | 2,099 | 1,552-2,838 | <0,001 | 2,179 | 1,066-4,455 | 0,03 |
|  |  | Myeloid hematological | 2,715 | 1,737-4,242 | <0,001 | , | , | , |
|  |  | Thyroid gland | 0,964 | 0,595-1,561 | 0,88 | 1,367 | 0,774-2,414 | 0,28 |
|  |  | Central nervous system | 0,587 | 0,289-1,192 | 0,140 | , | , | , |
| Primary treatment | Chemotherapy | No | REF |  |  | REF |  |  |
|  |  | Yes | 1,525 | 1,240-1,877 | <0,001 | 1,091 | 0,765-1,555 | 0,63 |
|  | Radiotherapy | No | REF |  |  | REF |  |  |
|  |  | Yes | 1,325 | 1,086-1,617 | 0,006 | 1,463 | 1,108-1,931 | 0,01 |
|  | Endocrine therapy | No | REF |  |  |  |  |  |
|  |  | Yes | 0,818 | 0,593-1,129 | 0,22 |  |  |  |
|  | Targeted therapy | No | REF |  |  |  |  |  |
|  |  | Yes | 0,996 | 0,687-1,446 | 0,99 |  |  |  |
|  | Surgery | No | REF |  |  | REF |  |  |
|  |  | Yes | 0,463 | 0,374-0,572 | <0,001 | 0,801 | 0,427-1,504 | 0,49 |
|  | Stem cell therapy | No | REF |  |  | REF |  |  |
|  |  | Yes | 2,864 | 1,930-4,250 | <0,001 | 0,708 | 0,257-1,951 | 0,50 |
| Tumor stage |  | I | REF |  |  | REF |  |  |
|  |  | II | 1,367 | 1,066-1,754 | 0,01 | 1,197 | 0,882-1,624 | 0,25 |
|  |  | III | 1,360 | 1,004-1,842 | 0,05 | 1,406 | 0,966-2,045 | 0,08 |
|  |  | IV | 1,517 | 0,956-2,408 | 0,08 | 1,214 | 0,701-2,103 | 0,49 |
| No multicollinearity was found for any of the independent variables [multicollinearity is indicated by: tolerance value <0.1, variance inflation factor value > 10 and variance proportions ≥0.7 on the same eigenvalue]; * Should be interpreted with caution due to wide 95% confidence intervals; REF: reference group; OR: odds ratio; CI: confidence interval; # Includes living with partner, parents, children, roommates or others. | | | | | | | | |

**Appendix Table 4g.**

|  |  |  | **Respiratory conditions (N= 3776)** | | | | | |
| --- | --- | --- | --- | --- | --- | --- | --- | --- |
|  |  |  | **Univariable logistic regression** | | | **Multivariable logistic regression** | | |
|  |  |  |  | | | **Nagelkerkes R^2^= 0,053** | | |
|  |  |  | **OR** | **95% CI** | **p-value** | **OR** | **95% CI** | **p-value** |
| Age at diagnosis in years |  | 18-24 years | REF |  |  | REF |  |  |
|  |  | 25-34 years | 0,763 | 0,579-1,006 | 0,06 | 0,765 | 0,554-1,056 | 0,10 |
|  |  | 35-39 years | 0,679 | 0,511-0,902 | 0,01 | 0,665 | 0,468-0,945 | 0,02 |
| Time since diagnosis in years |  | <11 years | REF |  |  | REF |  |  |
|  |  | 11-15 years | 1,407 | 1,107-1,786 | 0,005 | 1,655 | 1,267-2,161 | <0,001 |
|  |  | >15 years | 1,501 | 1,162-1,939 | 0,002 | 1,730 | 1,299-2,304 | <0,001 |
| Sex |  | Male | REF |  |  | REF |  |  |
|  |  | Female | 1,309 | 1,058-1,618 | 0,01 | 1,379 | 0,982-1,935 | 0,06 |
| Partner status at time of questionnaire |  | Partner | 0,779 | 0,603-1,006 | 0,06 | 0,780 | 0,585-1,039 | 0,09 |
|  |  | No partner | REF |  |  | REF |  |  |
| Education (achieved) |  | No education or primary education | 1,196 | 0,350-4,089 | 0,78 |  |  |  |
|  |  | Secondary education | 1,126 | 0,725-1,749 | 0,60 |  |  |  |
|  |  | Secondary vocational education | 1,114 | 0,846-1,466 | 0,44 |  |  |  |
|  |  | Higher (vocational) education | 1,056 | 0,798-1,396 | 0,71 |  |  |  |
|  |  | University education | REF |  |  |  |  |  |
| Living status |  | Living alone | 1,143 | 0,852-1,533 | 0,37 |  |  |  |
|  |  | Not living alone# | REF |  |  |  |  |  |
| Tumor type |  | Breast | REF |  |  | REF |  |  |
|  |  | Digestive track, other | 1,678 | 0,628-4,486 | 0,30 | 2,631 | 0,924-7,496 | 0,07 |
|  |  | Colon and rectal | 0,987 | 0,460-2,116 | 0,97 | 1,124 | 0,480-2,634 | 0,79 |
|  |  | Bone and soft tissue | 1,020 | 0,598-1,740 | 0,94 | 1,341 | 0,727-2,471 | 0,35 |
|  |  | Respiratory tract | 4,195 | 1,833-9,600 | <0,001 | 8,090 | 3,120-20,976 | <,001* |
|  |  | Melanoma | 0,722 | 0,441-1,183 | 0,20 | 1,151 | 0,605-2,190 | 0,67 |
|  |  | Other | 0,932 | 0,117-7,438 | 0,95 | 8,005 | 0,672-95,369 | 0,10* |
|  |  | Germ cell | 0,703 | 0,492-1,003 | 0,05 | 1,097 | 0,637-1,888 | 0,74 |
|  |  | Head and neck | 1,426 | 0,818-2,488 | 0,21 | 1,436 | 0,695-2,969 | 0,33 |
|  |  | Female genitalia | 0,909 | 0,616-1,343 | 0,63 | 1,160 | 0,712-1,891 | 0,55 |
|  |  | Male genitalia | 0,000 | 0,000 . | 0,999 | 0,000 | 0,000 . | 1,00 |
|  |  | Urinary tract | 0,430 | 0,102-1,810 | 0,25 | 0,699 | 0,158-3,097 | 0,64 |
|  |  | Lymphoid hematological | 1,754 | 1,293-2,380 | <0,001 | 1,517 | 0,746-3,082 | 0,25 |
|  |  | Myeloid hematological | 2,192 | 1,383-3,475 | <0,001 | , | , | , |
|  |  | Thyroid gland | 1,008 | 0,633-1,607 | 0,97 | 1,360 | 0,771-2,399 | 0,29 |
|  |  | Central nervous system | 0,626 | 0,318-1,232 | 0,18 | , | , | , |
| Primary treatment | Chemotherapy | No | REF |  |  | REF |  |  |
|  |  | Yes | 1,424 | 1,156-1,754 | <0,001 | 1,204 | 0,838-1,730 | 0,32 |
|  | Radiotherapy | No | REF |  |  | REF |  |  |
|  |  | Yes | 1,331 | 1,088-1,629 | 0,005 | 1,415 | 1,074-1,864 | 0,01 |
|  | Endocrine therapy | No | REF |  |  |  |  |  |
|  |  | Yes | 0,995 | 0,731-1,354 | 0,98 |  |  |  |
|  | Targeted therapy | No | REF |  |  |  |  |  |
|  |  | Yes | 0,926 | 0,629-1,364 | 0,70 |  |  |  |
|  | Surgery | No | REF |  |  | REF |  |  |
|  |  | Yes | 0,506 | 0,407-0,629 | <0,001 | 0,868 | 0,476-1,582 | 0,64 |
|  | Stem cell therapy | No | REF |  |  | REF |  |  |
|  |  | Yes | 2,409 | 1,592-3,644 | <0,001 | 0,637 | 0,214-1,893 | 0,42 |
| Tumor stage |  | I | REF |  |  | REF |  |  |
|  |  | II | 1,478 | 1,151-1,898 | 0,002 | 1,283 | 0,948-1,736 | 0,11 |
|  |  | III | 1,242 | 0,904-1,705 | 0,18 | 1,139 | 0,776-1,672 | 0,51 |
|  |  | IV | 1,833 | 1,176-2,858 | 0,007 | 1,524 | 0,903-2,573 | 0,11 |
| No multicollinearity was found for any of the independent variables [multicollinearity is indicated by: tolerance value <0.1, variance inflation factor value > 10 and variance proportions ≥0.7 on the same eigenvalue]; * Should be interpreted with caution due to wide 95% confidence intervals; REF: reference group; OR: odds ratio; CI: confidence interval; # Includes living with partner, parents, children, roommates or others. | | | | | | | | |

**Appendix Table 4h.**

|  |  |  | **Digestive conditions (N= 3776)** | | | | | |
| --- | --- | --- | --- | --- | --- | --- | --- | --- |
|  |  |  | **Univariable logistic regression** | | | **Multivariable logistic regression** | | |
|  |  |  |  | | | **Nagelkerkes R^2^= 0,050** | | |
|  |  |  | **OR** | **95% CI** | **p-value** | **OR** | **95% CI** | **p-value** |
| Age at diagnosis in years |  | 18-24 years | REF |  |  |  |  |  |
|  |  | 25-34 years | 0,913 | 0,701-1,188 | 0,50 |  |  |  |
|  |  | 35-39 years | 1,036 | 0,796-1,348 | 0,79 |  |  |  |
| Time since diagnosis in years |  | <11 years | REF |  |  | REF |  |  |
|  |  | 11-15 years | 1,222 | 0,988-1,512 | 0,07 | 1,372 | 1,086-1,734 | 0,01 |
|  |  | >15 years | 1,532 | 1,226-1,914 | <0,001 | 1,736 | 1,355-2,224 | <0,001 |
| Sex |  | Male | REF |  |  | REF |  |  |
|  |  | Female | 1,409 | 1,166-1,704 | <0,001 | 1,206 | 0,881-1,652 | 0,24 |
| Partner status at time of questionnaire |  | Partner | 0,824 | 0,654-1,038 | 0,10 |  |  |  |
|  |  | No partner | REF |  |  |  |  |  |
| Education (achieved) |  | No education or primary education | 1,404 | 0,471-4,188 | 0,54 | 1,425 | 0,455-4,464 | 0,54 |
|  |  | Secondary education | 1,241 | 0,830-1,853 | 0,29 | 0,915 | 0,571-1,467 | 0,71 |
|  |  | Secondary vocational education | 1,400 | 1,091-1,798 | 0,008 | 1,301 | 0,992-1,707 | 0,06 |
|  |  | Higher (vocational) education | 1,247 | 0,965-1,611 | 0,09 | 1,205 | 0,914-1,588 | 0,19 |
|  |  | University education | REF |  |  | REF |  |  |
| Living status |  | Living alone | 1,223 | 0,946-1,581 | 0,13 |  |  |  |
|  |  | Not living alone# | REF |  |  |  |  |  |
| Tumor type |  | Breast | REF |  |  | REF |  |  |
|  |  | Digestive track, other | 2,679 | 1,199-5,985 | 0,02 | 3,639 | 1,536-8,622 | 0,003 |
|  |  | Colon and rectal | 2,886 | 1,716-4,852 | <0,001 | 3,382 | 1,911-5,984 | <0,001 |
|  |  | Bone and soft tissue | 0,713 | 0,417-1,220 | 0,22 | 0,711 | 0,381-1,329 | 0,29 |
|  |  | Respiratory tract | 1,421 | 0,528-3,822 | 0,49 | 2,053 | 0,711-5,929 | 0,18 |
|  |  | Melanoma | 0,859 | 0,567-1,301 | 0,47 | 1,219 | 0,758-1,961 | 0,41 |
|  |  | Other | 1,563 | 0,328-7,447 | 0,58 | 0,000 | ,000 . | 0,999 |
|  |  | Germ cell | 0,754 | 0,552-1,029 | 0,08 | 1,071 | 0,674-1,702 | 0,77 |
|  |  | Head and neck | 0,990 | 0,565-1,735 | 0,97 | 0,950 | 0,505-1,788 | 0,87 |
|  |  | Female genitalia | 1,764 | 1,305-2,385 | <0,001 | 1,987 | 1,379-2,865 | <0,001 |
|  |  | Male genitalia | 0,000 | 0,000 . | 0,999 | 0,000 | ,000 . | 0,999 |
|  |  | Urinary tract | 1,287 | 0,558-2,968 | 0,55 | 1,739 | 0,725-4,174 | 0,22 |
|  |  | Lymphoid hematological | 1,226 | 0,914-1,645 | 0,17 | 0,992 | 0,543-1,814 | 0,98 |
|  |  | Myeloid hematological | 1,633 | 1,041-2,564 | 0,03 | , | , | , |
|  |  | Thyroid gland | 0,959 | 0,628-1,465 | 0,85 | 1,019 | 0,645-1,611 | 0,94 |
|  |  | Central nervous system | 1,008 | 0,606-1,678 | 0,97 | , | , | , |
| Primary treatment | Chemotherapy | No | REF |  |  |  |  |  |
|  |  | Yes | 1,094 | 0,913-1,311 | 0,33 |  |  |  |
|  | Radiotherapy | No | REF |  |  | REF |  |  |
|  |  | Yes | 1,225 | 1,024-1,465 | 0,03 | 1,347 | 1,058-1,714 | 0,02 |
|  | Endocrine therapy | No | REF |  |  |  |  |  |
|  |  | Yes | 1,099 | 0,842-1,434 | 0,49 |  |  |  |
|  | Targeted therapy | No | REF |  |  |  |  |  |
|  |  | Yes | 0,813 | 0,569-1,162 | 0,26 |  |  |  |
|  | Surgery | No | REF |  |  | REF |  |  |
|  |  | Yes | 0,726 | 0,592-0,891 | 0,002 | 0,754 | 0,458-1,241 | 0,27 |
|  | Stem cell therapy | No | REF |  |  | REF |  |  |
|  |  | Yes | 1,865 | 1,247-2,790 | 0,002 | 0,445 | 0,131-1,510 | 0,19 |
| Tumor stage |  | I | REF |  |  | REF |  |  |
|  |  | II | 1,082 | 0,867-1,352 | 0,49 | 1,138 | 0,884-1,465 | 0,32 |
|  |  | III | 1,009 | 0,763-1,333 | 0,95 | 1,048 | 0,768-1,430 | 0,77 |
|  |  | IV | 1,480 | 0,992-2,209 | 0,06 | 1,542 | 0,969-2,454 | 0,07 |
| No multicollinearity was found for any of the independent variables [multicollinearity is indicated by: tolerance value <0.1, variance inflation factor value > 10 and variance proportions ≥0.7 on the same eigenvalue]; * Should be interpreted with caution due to wide 95% confidence intervals; REF: reference group; OR: odds ratio; CI: confidence interval; # Includes living with partner, parents, children, roommates or others. | | | | | | | | |

**Appendix Table 4i.**

|  |  |  | **Rheumatoid arthritis (N= 3776)** | | | | | |
| --- | --- | --- | --- | --- | --- | --- | --- | --- |
|  |  |  | **Univariable logistic regression** | | | **Multivariable logistic regression** | | |
|  |  |  |  | | | **Nagelkerkes R^2^= 0,064** | | |
|  |  |  | **OR** | **95% CI** | **p-value** | **OR** | **95% CI** | **p-value** |
| Age at diagnosis in years |  | 18-24 years | REF |  |  |  |  |  |
|  |  | 25-34 years | 0,880 | 0,447-1,730 | 0,71 |  |  |  |
|  |  | 35-39 years | 1,186 | 0,614-2,291 | 0,61 |  |  |  |
| Time since diagnosis in years |  | <11 years | REF |  |  | REF |  |  |
|  |  | 11-15 years | 1,656 | 0,954-2,875 | 0,07 | 1,700 | 0,973-2,970 | 0,06 |
|  |  | >15 years | 1,925 | 1,085-3,416 | 0,03 | 1,958 | 1,092-3,508 | 0,02 |
| Sex |  | Male | REF |  |  | REF |  |  |
|  |  | Female | 2,192 | 1,290-3,723 | 0,00 | 2,000 | 0,924-4,331 | 0,08 |
| Partner status at time of questionnaire |  | Partner | 0,838 | 0,475-1,481 | 0,54 |  |  |  |
|  |  | No partner | REF |  |  |  |  |  |
| Education (achieved) |  | No education or primary education | 0,000 | 0,000 . | 0,998 | 0,000 | ,000 . | 1,00 |
|  |  | Secondary education | 2,608 | 0,962-7,076 | 0,06 | 2,619 | 0,955-7,180 | 0,06 |
|  |  | Secondary vocational education | 2,401 | 1,148-5,021 | 0,02 | 2,388 | 1,135-5,027 | 0,02 |
|  |  | Higher (vocational) education | 2,040 | 0,958-4,346 | 0,07 | 1,997 | 0,934-4,271 | 0,08 |
|  |  | University education | REF |  |  | REF |  |  |
| Living status |  | Living alone | 1,017 | 0,520-1,989 | 0,96 |  |  |  |
|  |  | Not living alone# | REF |  |  |  |  |  |
| Tumor type |  | Breast | REF |  |  | REF |  |  |
|  |  | Digestive track, other | 0,000 | ,000 . | ,998 | 0,000 | ,000 . | 0,998 |
|  |  | Colon and rectal | 0,000 | ,000 . | ,997 | 0,000 | ,000 . | 0,997 |
|  |  | Bone and soft tissue | 0,391 | 0,092-1,659 | 0,20 | 0,453 | 0,103-1,990 | 0,29 |
|  |  | Respiratory tract | 1,232 | 0,161-9,417 | 0,84 | 1,847 | 0,230-14,823 | 0,56* |
|  |  | Melanoma | 0,742 | 0,303-1,817 | 0,51 | 0,877 | 0,321-2,396 | 0,80 |
|  |  | Other | 0,000 | ,000 . | 0,999 | 0,000 | ,000 . | 0,999 |
|  |  | Germ cell | 0,443 | 0,207-0,948 | 0,04 | 0,819 | 0,270-2,483 | 0,73 |
|  |  | Head and neck | 0,276 | 0,037-2,052 | 0,21 | 0,344 | 0,045-2,624 | 0,30 |
|  |  | Female genitalia | 0,477 | 0,195-1,164 | 0,10 | 0,441 | 0,173-1,123 | 0,09 |
|  |  | Male genitalia | 0,000 | ,000 . | 0,999 | 0,000 | ,000 . | 0,999 |
|  |  | Urinary tract | 0,801 | 0,106-6,043 | 0,83 | 1,016 | 0,128-8,087 | 0,99 |
|  |  | Lymphoid hematological | 0,641 | 0,315-1,302 | 0,22 | 0,871 | 0,398-1,904 | 0,73 |
|  |  | Myeloid hematological | 0,230 | 0,031-1,710 | 0,15 | 0,256 | 0,033-1,994 | 0,19 |
|  |  | Thyroid gland | 1,893 | 0,961-3,729 | 0,07 | 2,254 | 1,125-4,516 | 0,02 |
|  |  | Central nervous system | 0,224 | 0,030-1,662 | 0,14 | 0,302 | 0,040-2,289 | 0,25 |
| Primary treatment | Chemotherapy | No | REF |  |  |  |  |  |
|  |  | Yes | 0,880 | 0,563-1,376 | 0,58 |  |  |  |
|  | Radiotherapy | No | REF |  |  | REF |  |  |
|  |  | Yes | 1,466 | 0,935-2,300 | 0,096 | 0,942 | 0,543-1,632 | 0,83 |
|  | Endocrine therapy | No | REF |  |  |  |  |  |
|  |  | Yes | 1,430 | 0,783-2,614 | 0,25 |  |  |  |
|  | Targeted therapy | No | REF |  |  |  |  |  |
|  |  | Yes | 0,633 | 0,230-1,743 | 0,38 |  |  |  |
|  | Surgery | No | REF |  |  |  |  |  |
|  |  | Yes | 1,108 | 0,637-1,929 | 0,72 |  |  |  |
|  | Stem cell therapy | No | REF |  |  |  |  |  |
|  |  | Yes | 0,690 | 0,168-2,838 | 0,61 |  |  |  |
| Tumor stage |  | I | REF |  |  |  |  |  |
|  |  | II | 0,685 | 0,392-1,197 | 0,18 |  |  |  |
|  |  | III | 0,708 | 0,353-1,421 | 0,33 |  |  |  |
|  |  | IV | 0,000 | ,000 . | 0,995 |  |  |  |
| No multicollinearity was found for any of the independent variables [multicollinearity is indicated by: tolerance value <0.1, variance inflation factor value > 10 and variance proportions ≥0.7 on the same eigenvalue]; * Should be interpreted with caution due to wide 95% confidence intervals; REF: reference group; OR: odds ratio; CI: confidence interval; # Includes living with partner, parents, children, roommates or others. | | | | | | | | |

**Appendix Table 4j.**

|  |  |  | **Arthrosis (N= 3776)** | | | | | |
| --- | --- | --- | --- | --- | --- | --- | --- | --- |
|  |  |  | **Univariable logistic regression** | | | **Multivariable logistic regression** | | |
|  |  |  |  | | | **Nagelkerkes R^2^= 0,101** | | |
|  |  |  | **OR** | **95% CI** | **p-value** | **OR** | **95% CI** | **p-value** |
| Age at diagnosis in years |  | 18-24 years | REF |  |  | REF |  |  |
|  |  | 25-34 years | 1,702 | 1,014-2,856 | 0,04 | 1,486 | 0,872-2,533 | 0,15 |
|  |  | 35-39 years | 3,637 | 2,212-5,979 | <0,001 | 2,902 | 1,709-4,926 | <0,001 |
| Time since diagnosis in years |  | <11 years | REF |  |  | REF |  |  |
|  |  | 11-15 years | 2,350 | 1,677-3,294 | <0,001 | 2,335 | 1,657-3,291 | <0,001 |
|  |  | >15 years | 3,318 | 2,361-4,661 | <0,001 | 3,376 | 2,383-4,783 | <0,001 |
| Sex |  | Male | REF |  |  | REF |  |  |
|  |  | Female | 1,965 | 1,475 2,619 | <0,001 | 1,429 | 0,937-2,180 | 0,10 |
| Partner status at time of questionnaire |  | Partner | 0,866 | 0,625-1,200 | 0,39 |  |  |  |
|  |  | No partner | REF |  |  |  |  |  |
| Education (achieved) |  | No education or primary education | 0,000 | 0,000 . | 0,998 | 0,000 | ,000 . | 0,998 |
|  |  | Secondary education | 2,387 | 1,360-4,188 | 0,002 | 1,870 | 1,050-3,330 | 0,03 |
|  |  | Secondary vocational education | 2,122 | 1,415-3,180 | <0,001 | 1,888 | 1,250-2,852 | 0,00 |
|  |  | Higher (vocational) education | 2,113 | 1,405-3,178 | <0,001 | 2,023 | 1,336-3,063 | <0,001 |
|  |  | University education | REF |  |  | REF |  |  |
| Living status |  | Living alone | 1,173 | 0,816-1,684 | 0,39 |  |  |  |
|  |  | Not living alone# | REF |  |  |  |  |  |
| Tumor type |  | Breast | REF |  |  | REF |  |  |
|  |  | Digestive track, other | 0,000 | 0,000 | . | 0,000 | ,000 . | 0,998 |
|  |  | Colon and rectal | 0,477 | 0,170-1,336 | 0,16 | 0,674 | 0,226-2,009 | 0,48 |
|  |  | Bone and soft tissue | 0,322 | 0,139-0,748 | 0,008 | 0,469 | 0,190-1,161 | 0,10 |
|  |  | Respiratory tract | 1,074 | 0,317-3,635 | 0,91 | 2,223 | 0,603-8,195 | 0,23 |
|  |  | Melanoma | 0,589 | 0,344-1,007 | 0,05 | 0,785 | 0,411-1,502 | 0,47 |
|  |  | Other | 0,000 | 0,000 . | 0,999 | 0,000 | ,000 . | 0,999 |
|  |  | Germ cell | 0,352 | 0,225-0,551 | <0,001 | 0,643 | 0,326-1,270 | 0,20 |
|  |  | Head and neck | 0,631 | 0,298-1,334 | 0,23 | 0,842 | 0,369-1,926 | 0,69 |
|  |  | Female genitalia | 0,779 | 0,516-1,176 | 0,23 | 0,859 | 0,520-1,417 | 0,55 |
|  |  | Male genitalia | 2,148 | 0,238-19,419 | 0,50 | 2,306 | 0,228-23,298 | 0,48* |
|  |  | Urinary tract | 0,441 | 0,105-1,854 | 0,26 | 0,664 | 0,149-2,962 | 0,59 |
|  |  | Lymphoid hematological | 0,468 | 0,304-0,721 | <0,001 | 0,816 | 0,468-1,421 | 0,47 |
|  |  | Myeloid hematological | 1,031 | 0,579-1,836 | 0,92 | 1,386 | 0,696-2,760 | 0,35 |
|  |  | Thyroid gland | 0,676 | 0,395-1,158 | 0,15 | 0,959 | 0,525-1,753 | 0,89 |
|  |  | Central nervous system | 0,374 | 0,160-0,870 | 0,02 | 0,630 | 0,254-1,562 | 0,32 |
| Primary treatment | Chemotherapy | No | REF |  |  |  |  |  |
|  |  | Yes | 1,037 | 0,804-1,337 | 0,78 |  |  |  |
|  | Radiotherapy | No | REF |  |  | REF |  |  |
|  |  | Yes | 1,252 | 0,974-1,611 | 0,08 | 0,957 | 0,702-1,304 | 0,78 |
|  | Endocrine therapy | No | REF |  |  | REF |  |  |
|  |  | Yes | 1,743 | 1,257-2,418 | <0,001 | 1,081 | 0,699-1,672 | 0,73 |
|  | Targeted therapy | No | REF |  |  |  |  |  |
|  |  | Yes | 0,823 | 0,496-1,366 | 0,45 |  |  |  |
|  | Surgery | No | REF |  |  |  |  |  |
|  |  | Yes | 1,110 | 0,812-1,516 | 0,51 |  |  |  |
|  | Stem cell therapy | No | REF |  |  |  |  |  |
|  |  | Yes | 1,575 | 0,892-2,778 | 0,12 |  |  |  |
| Tumor stage |  | I | REF |  |  |  |  |  |
|  |  | II | 1,061 | 0,785-1,433 | 0,70 |  |  |  |
|  |  | III | 0,708 | 0,463-1,083 | 0,11 |  |  |  |
|  |  | IV | 0,545 | 0,250-1,188 | 0,13 |  |  |  |
| No multicollinearity was found for any of the independent variables [multicollinearity is indicated by: tolerance value <0.1, variance inflation factor value > 10 and variance proportions ≥0.7 on the same eigenvalue]; * Should be interpreted with caution due to wide 95% confidence intervals; REF: reference group; OR: odds ratio; CI: confidence interval; # Includes living with partner, parents, children, roommates or others. | | | | | | | | |

**Appendix Table 4k.**

|  |  |  | **Depression (N= 3776)** | | | | | |
| --- | --- | --- | --- | --- | --- | --- | --- | --- |
|  |  |  | **Univariable logistic regression** | | | **Multivariable logistic regression** | | |
|  |  |  |  | | | **Nagelkerkes R^2^= 0,044** | | |
|  |  |  | **OR** | **95% CI** | **p-value** | **OR** | **95% CI** | **p-value** |
| Age at diagnosis in years |  | 18-24 years | REF |  |  | REF |  |  |
|  |  | 25-34 years | 0,679 | 0,500-0,922 | 0,01 | 0,636 | 0,459-0,882 | 0,01 |
|  |  | 35-39 years | 0,627 | 0,458-0,857 | 0,003 | 0,557 | 0,392-0,791 | 0,001 |
| Time since diagnosis in years |  | <11 years | REF |  |  |  |  |  |
|  |  | 11-15 years | 0,926 | 0,707-1,212 | 0,57 |  |  |  |
|  |  | >15 years | 1,138 | 0,859-1,508 | 0,37 |  |  |  |
| Sex |  | Male | REF |  |  | REF |  |  |
|  |  | Female | 1,330 | 1,045-1,693 | 0,02 | 1,223 | 0,864-1,730 | 0,26 |
| Partner status at time of questionnaire |  | Partner | 0,528 | 0,405-0,688 | <0,001 | 0,590 | 0,415-0,838 | 0,003 |
|  |  | No partner | REF |  |  | REF |  |  |
| Education (achieved) |  | No education or primary education | 1,367 | 0,313-5,973 | 0,68 | 1,310 | 0,291-5,894 | 0,73 |
|  |  | Secondary education | 1,649 | 1,001-2,717 | 0,05 | 1,495 | 0,893-2,502 | 0,13 |
|  |  | Secondary vocational education | 1,656 | 1,188-2,308 | 0,003 | 1,634 | 1,166-2,289 | 0,004 |
|  |  | Higher (vocational) education | 1,407 | 0,999-1,980 | 0,05 | 1,402 | 0,992-1,981 | 0,06 |
|  |  | University education | REF |  |  | REF |  |  |
| Living status |  | Living alone | 1,714 | 1,274-2,307 | <0,001 | 1,163 | 0,782 1,730 | 0,46 |
|  |  | Not living alone# | REF |  |  |  |  |  |
| Tumor type |  | Breast | REF |  |  | REF |  |  |
|  |  | Digestive track, other | 0,355 | 0,048-2,640 | 0,31 | 0,422 | 0,056-3,199 | 0,40 |
|  |  | Colon and rectal | 1,211 | 0,562-2,610 | 0,63 | 1,286 | 0,578-2,863 | 0,54 |
|  |  | Bone and soft tissue | 1,655 | 1,007-2,721 | 0,05 | 1,379 | 0,798-2,385 | 0,25 |
|  |  | Respiratory tract | 0,396 | 0,053-2,956 | 0,37 | 0,359 | 0,047-2,736 | 0,32 |
|  |  | Melanoma | 0,531 | 0,290-0,971 | 0,04 | 0,522 | 0,281-0,967 | 0,04 |
|  |  | Other | 1,143 | 0,143-9,142 | 0,899 | 1,166 | 0,144-9,466 | 0,89 |
|  |  | Germ cell | 0,699 | 0,474-1,032 | 0,07 | 0,711 | 0,415-1,217 | 0,21 |
|  |  | Head and neck | 0,755 | 0,355-1,606 | 0,47 | 0,677 | 0,307-1,495 | 0,34 |
|  |  | Female genitalia | 1,433 | 0,985-2,086 | 0,06 | 1,280 | 0,864-1,894 | 0,22 |
|  |  | Male genitalia | 0,000 | 0,000 . | 0,999 | 0,000 | 0,000 . | 0,999 |
|  |  | Urinary tract | 1,764 | 0,720-4,323 | 0,21 | 1,664 | 0,661-4,188 | 0,28 |
|  |  | Lymphoid hematological | 1,187 | 0,831-1,695 | 0,35 | 0,813 | 0,411-1,607 | 0,55 |
|  |  | Myeloid hematological | 0,965 | 0,511-1,821 | 0,91 | 0,648 | 0,276-1,523 | 0,32 |
|  |  | Thyroid gland | 0,759 | 0,434-1,325 | 0,33 | 0,721 | 0,408-1,276 | 0,26 |
|  |  | Central nervous system | 0,526 | 0,238-1,163 | 0,11 | 0,449 | 0,198-1,018 | 0,06 |
| Primary treatment | Chemotherapy | No | REF |  |  |  |  |  |
|  |  | Yes | 1,135 | 0,901-1,430 | 0,28 |  |  |  |
|  | Radiotherapy | No | REF |  |  |  |  |  |
|  |  | Yes | 0,989 | 0,787-1,242 | 0,92 |  |  |  |
|  | Endocrine therapy | No | REF |  |  |  |  |  |
|  |  | Yes | 1,075 | 0,766-1,511 | 0,68 |  |  |  |
|  | Targeted therapy | No | REF |  |  |  |  |  |
|  |  | Yes | 1,191 | 0,797-1,780 | 0,39 |  |  |  |
|  | Surgery | No | REF |  |  | REF |  |  |
|  |  | Yes | 0,761 | 0,587-0,986 | 0,04 | 0,756 | 0,437-1,307 | 0,32 |
|  | Stem cell therapy | No | REF |  |  |  |  |  |
|  |  | Yes | 0,745 | 0,375-1,478 | 0,40 |  |  |  |
| Tumor stage |  | I | REF |  |  |  |  |  |
|  |  | II | 1,082 | 0,825-1,420 | 0,57 |  |  |  |
|  |  | III | 0,841 | 0,586-1,207 | 0,35 |  |  |  |
|  |  | IV | 0,761 | 0,413-1,403 | 0,38 |  |  |  |
| No multicollinearity was found for any of the independent variables [multicollinearity is indicated by: tolerance value <0.1, variance inflation factor value > 10 and variance proportions ≥0.7 on the same eigenvalue]; * Should be interpreted with caution due to wide 95% confidence intervals; REF: reference group; OR: odds ratio; CI: confidence interval; # Includes living with partner, parents, children, roommates or others. | | | | | | | | |

**Appendix Table 4l.**

|  |  |  | **Secondary malignancy^$^ (N= 3776)** | | | | | |
| --- | --- | --- | --- | --- | --- | --- | --- | --- |
|  |  |  | **Univariable logistic regression** | | | **Multivariable logistic regression** | | |
|  |  |  |  | | | **Nagelkerkes R^2^= 0,075** | | |
|  |  |  | OR | 95% CI | p-value | **OR** | **95% CI** | **p-value** |
| Age at diagnosis in years |  | 18-24 years | REF |  |  | REF |  |  |
|  |  | 25-34 years | 1,336 | 0,857-2,081 | 0,20 | 1,389 | 0,868-2,222 | 0,17 |
|  |  | 35-39 years | 1,801 | 1,165-2,785 | 0,008 | 1,800 | 1,113-2,911 | 0,02 |
| Time since diagnosis in years |  | <11 years | REF |  |  | REF |  |  |
|  |  | 11-15 years | 2,006 | 1,406-2,862 | <0,001 | 2,110 | 1,471-3,026 | <0,001 |
|  |  | >15 years | 3,597 | 2,547-5,079 | <0,001 | 3,761 | 2,640-5,358 | <0,001 |
| Sex |  | Male | REF |  |  | REF |  |  |
|  |  | Female | 1,473 | 1,111-1,952 | 0,007 | 1,158 | 0,784-1,712 | 0,46 |
| Partner status at time of questionnaire |  | Partner | 0,738 | 0,533-1,020 | 0,07 | 0,831 | 0,524-1,319 | 0,43 |
|  |  | No partner | REF |  |  | REF |  |  |
| Education (achieved) |  | No education or primary education | 0,800 | 0,106-6,062 | 0,83 | 0,535 | 0,065-4,420 | 0,56 |
|  |  | Secondary education | 1,078 | 0,580-2,004 | 0,81 | 0,853 | 0,451-1,613 | 0,63 |
|  |  | Secondary vocational education | 1,370 | 0,945-1,985 | 0,097 | 1,182 | 0,808-1,730 | 0,39 |
|  |  | Higher (vocational) education | 1,351 | 0,928-1,967 | 0,12 | 1,283 | 0,875-1,882 | 0,20 |
|  |  | University education | REF |  |  | REF |  |  |
| Living status |  | Living alone | 1,441 | 1,011-2,053 | 0,04 |  |  |  |
|  |  | Not living alone# | REF |  |  |  |  |  |
| Tumor type |  | Breast | REF |  |  | REF |  |  |
|  |  | Digestive track, other | 0,399 | 0,054-2,970 | 0,37 | 0,469 | 0,061-3,632 | 0,47 |
|  |  | Colon and rectal | 0,814 | 0,319-2,082 | 0,67 | 1,055 | 0,396-2,812 | 0,92 |
|  |  | Bone and soft tissue | 0,741 | 0,374-1,469 | 0,39 | 0,803 | 0,376-1,713 | 0,57 |
|  |  | Respiratory tract | 2,011 | 0,677-5,976 | 0,21 | 3,122 | 0,963-10,117 | 0,06* |
|  |  | Melanoma | 0,743 | 0,424-1,302 | 0,30 | 0,881 | 0,467-1,662 | 0,70 |
|  |  | Other | 0,000 | 0,000 . | 0,999 | 0,000 | 0,000 . | 0,999 |
|  |  | Germ cell | 0,380 | 0,231-0,625 | <0,001 | 0,491 | 0,253-0,951 | 0,04 |
|  |  | Head and neck | 0,409 | 0,147-1,142 | 0,09 | 0,422 | 0,145-1,229 | 0,11 |
|  |  | Female genitalia | 0,753 | 0,470-1,206 | 0,24 | 0,769 | 0,456-1,297 | 0,33 |
|  |  | Male genitalia | 0,000 | 0,000 . | 0,999 | 0,000 | 0,000 . | 0,999 |
|  |  | Urinary tract | 1,250 | 0,433-3,607 | 0,68 | 1,869 | 0,616-5,666 | 0,27 |
|  |  | Lymphoid hematological | 1,033 | 0,701-1,521 | 0,87 | 0,892 | 0,379-2,100 | 0,79 |
|  |  | Myeloid hematological | 0,986 | 0,509-1,911 | 0,97 | 0,675 | 0,231-1,973 | 0,47 |
|  |  | Thyroid gland | 0,853 | 0,486-1,497 | 0,58 | 0,974 | 0,537-1,766 | 0,93 |
|  |  | Central nervous system | 0,591 | 0,266-1,311 | 0,20 | 0,800 | 0,348-1,841 | 0,60 |
| Primary treatment | Chemotherapy | No | REF |  |  |  |  |  |
|  |  | Yes | 1,170 | 0,896-1,528 | 0,25 |  |  |  |
|  | Radiotherapy | No | REF |  |  | REF |  |  |
|  |  | Yes | 1,288 | 0,990-1,675 | 0,06 | 1,088 | 0,788-1,502 | 0,61 |
|  | Endocrine therapy | No | REF |  |  |  |  |  |
|  |  | Yes | 1,210 | 0,830-1,763 | 0,32 |  |  |  |
|  | Targeted therapy | No | REF |  |  |  |  |  |
|  |  | Yes | 1,297 | 0,829-2,030 | 0,25 |  |  |  |
|  | Surgery | No | REF |  |  | REF |  |  |
|  |  | Yes | 0,720 | 0,536-0,966 | 0,03 | 0,721 | 0,355-1,464 | 0,37 |
|  | Stem cell therapy | No | REF |  |  | REF |  |  |
|  |  | Yes | 2,041 | 1,190-3,498 | 0,01 | 1,708 | 0,895-3,261 | 0,11 |
| Tumor stage |  | I | REF |  |  |  |  |  |
|  |  | II | 1,092 | 0,787-1,514 | 0,56 |  |  |  |
|  |  | III | 1,010 | 0,668-1,526 | 0,96 |  |  |  |
|  |  | IV | 1,193 | 0,640-2,221 | 0,58 |  |  |  |
| No multicollinearity was found for any of the independent variables [multicollinearity is indicated by: tolerance value <0.1, variance inflation factor value > 10 and variance proportions ≥0.7 on the same eigenvalue]; * Should be interpreted with caution due to wide 95% confidence intervals; REF: reference group; OR: odds ratio; CI: confidence interval; # Includes living with partner, parents, children, roommates or others. $; The number of AYAs who are diagnosed with a new primary might be underestimated as we have recategorised and added the answering option 'I don't know' to 'No'. | | | | | | | | |
